# Supplementary material for: Direct oxidative carbonylation of methane to acetic acid via high-valent iron-oxo mediated water activation
Source: Nat Commun. 2026 Mar 7;17:3644. doi: 10.1038/s41467-026-70339-w (PMC13096338; doi:10.1038/s41467-026-70339-w)
Supplement: Supplementary file 1 — Supplementary Information [file 41467_2026_70339_MOESM1_ESM.pdf]

Supplementary Information

Direct oxidative carbonylation of methane to acetic acid via high-valent iron-oxo mediated water activation

Haonan Zhang[1]†, Richard J. Lewis[2]†, A. Iulian Dugulan[3]†, Yang Li[1], Shuai Wang[1], Zhenxing Wang[4], Jianrong Zeng[5], Nicholas F. Dummer[2], Yanyan Xi[1], Yunyun Li[1], Thomas E. Davies[2], Mingbo Wu[1,6*], Graham J. Hutchings[2*,] Wenting Wu[1*]

[1] State Key Laboratory of Heavy Oil Processing, College of Chemistry and Chemical Engineering, China University of Petroleum (East China), Qingdao 266580, P. R. China

[2] Max Planck-Cardiff Centre on the Fundamentals of Heterogeneous Catalysis FUNCAT, Cardiff Catalysis Institute, School of Chemistry, Cardiff University, Main Building, Park Place, Cardiff CF10 3AT, UK.

[3] Fundamental Aspects of Materials and Energy (FAME), Department of Radiation Science and Technology (RST), Delft University of Technology, Mekelweg 15, 2629 JB, Delft, the Netherlands.

[4] Wuhan National High Magnetic Field Center, Huazhong University of Science and Technology, Wuhan 430074, China

[5] Shanghai Synchrotron Radiation Facility, Shanghai Advanced Research Institute, Chinese Academy of Sciences, Shanghai 201204, China

[6] State Key Laboratory of Advanced Optical Polymer and Manufacturing Technology, College of Chemical Engineering, Qingdao University of Science & Technology, Qingdao 266061, P. R. China.

*Corresponding author. E-mail: wuwt@upc.edu.cn (W. T. W.); hutch@cardiff.ac.uk (G.J.H.); wumb@upc.edu.cn (M. B.W.)

† Haonan Zhang, Richard J. Lewis, and A. Iulian Dugulan contributed equally to this work.

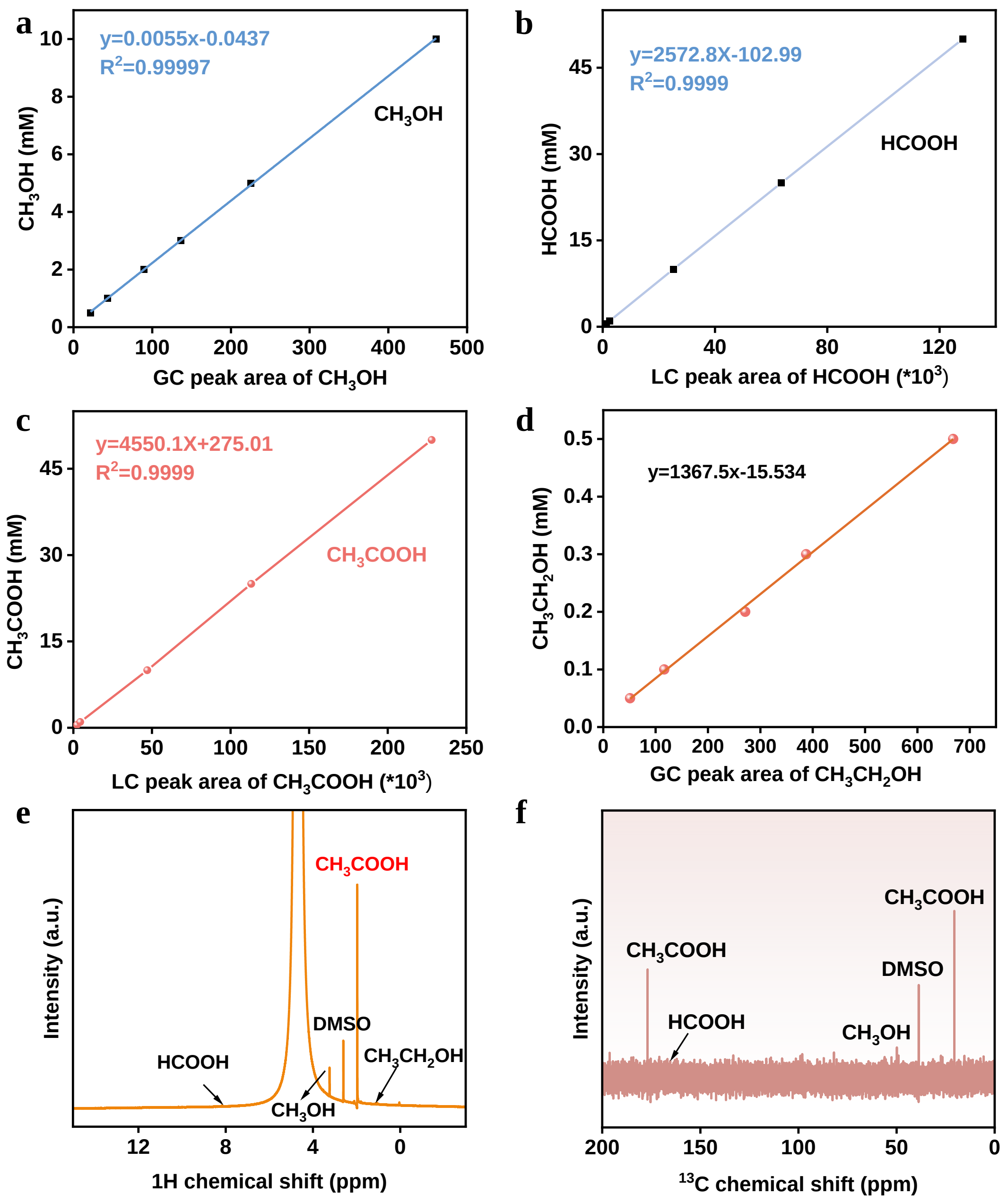

Supplementary Figure 1. (**a**) Calibration curve for quantification of CH_3OH by gas chromatography. (**b**) Calibration curve for quantification of HCOOH by liquid chromatography. (**c**) Calibration curve for quantification of CH_3COOH by liquid chromatography. (**d**) Calibration curve for quantification of CH_3CH_2OH by gas chromatography. (**e-f**) ^{1}H-NMR and ^{13}C-NMR spectra of liquid products, reaction conditions: 463 K, 10 mg catalyst, 2 h, 20 mL H_2O, 30 bar CH_4, 3 bar O_2, 6 bar CO.

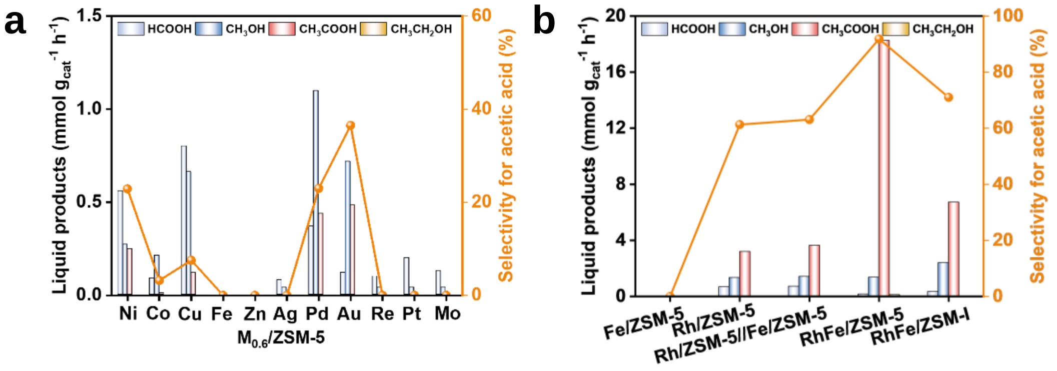

Supplementary Figure 2. The performance of different metal in ZSM-5. Reaction conditions: 10 mg catalyst, 3 h, 20 mL H_2O, 30 bar CH_4, 3 bar O_2, 6 bar CO. The Rh/ZSM-5//Fe/ZSM-5 catalyst was prepared by mixing 10 mg Rh/ZSM-5 and 10 mg Fe/ZSM-5.

RhFe/ZSM-I: Fe/ZSM-5 was first synthesized via the in-situ seed method, followed by the introduction of Rh species through impregnation and subsequent calcination at 550°C, yielding a catalyst with spatially separated Rh/Fe sites (denoted as RhFe/ZSM-I).

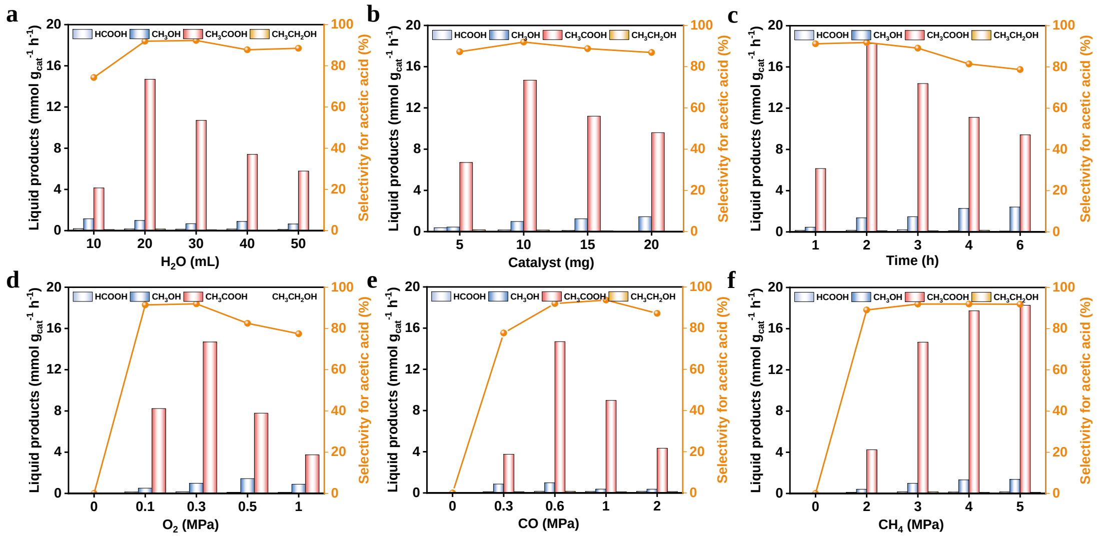

Supplementary Figure 3. Catalytic performance evaluation of methane oxidative. (**a**) Different H_2O volume in the reaction, reaction conditions: 10 mg catalyst, 3 h, 20 mL H_2O, 30 bar CH_4, 3 bar O_2, 6 bar CO; (**b**) Different amount of RhFe/ZSM-5 catalyst, reaction conditions: 10 mg catalyst, 3 h, 20 mL H_2O, 30 bar CH_4, 3 bar O_2, 6 bar CO; (**c**) Different reaction time, reaction conditions: 10 mg catalyst, 2 h, 20 mL H_2O, 40 bar CH_4, 3 bar O_2, 6 bar CO; (**d**) Different O_2 pressure, 10 mg catalyst, 2 h, 20 mL H_2O, 30 bar CH_4, 6 bar CO, Ar as balance gas; (**e**) Different CO pressure, 10 mg catalyst, 2 h, 20 mL H_2O, 30 bar CH_4, 3 bar O_2; (**f**) Different CH_4 pressure, 10 mg catalyst, 2 h, 20 mL H_2O, 3 bar O_2, 6 bar CO, 463 K.

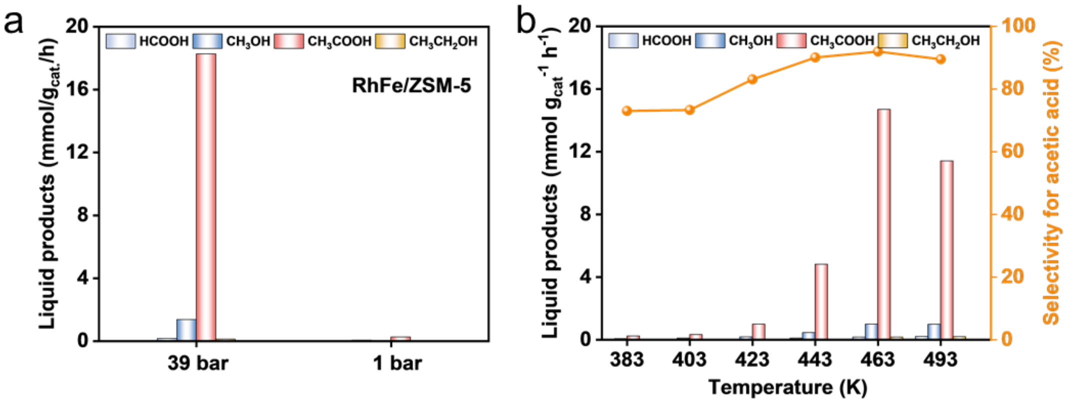

Supplementary Figure 4. (**a**) Catalytic performance at different pressure, reaction conditions: 10 mg catalyst, 2 h, 20 mL H_2O, $CH_4/O_2/CO$=10/1/2. (**b**) Catalytic performance at different temperature, reaction conditions: 10 mg catalyst, 3 h, 20 mL H_2O, 30 bar CH_4, 3 bar O_2, 6 bar CO.

H_2O is another key factor for catalytic methane oxidation carbonylation (Supplementary Figure 3a). With the H_2O volume increasing, the yield of oxygenates increases gradually and levels off after 20 mL. This result is attributed to the fact that H_2O can promote the dissolution of methane and facilitate the desorption of CH_3OH, HCOOH, and CH_3COOH. When the H_2O volume exceeds 20 mL, the yield of oxygenates is limited by the number of available active sites in the catalyst. Finally, the effect of the amount of catalyst and reaction time is explored (Supplementary Figure 3b-c). The amount of CH_3COOH increases gradually with the reaction catalysts and time. Upon the reaction for 2 h, the highest productivity of acetic acid was achieved with 10 mg catalyst.

Noteworthily, methane oxidation is a temperature-sensitive reaction, the productivity and selectivity of acetic acid show that the volcanic trend and the highest values are achieved at 463 K (Supplementary Figure 4). No products are formed in the absence of O_2 and CO (Supplementary Figure 3d-e), suggesting that O_2 activation is a critical step, CO is involved in the C-C coupling process. The highest productivity of acetic acid was achieved at 3 bar O_2 and 6 bar CO for FeRh/ZSM-5. High pressure is beneficial to increase the solubility of methane in water and promote the reaction (Supplementary Figure 3f).

When the reaction was carried out at atmospheric pressure (Supplementary Figure 4a), the RhFe/ZSM-5 catalyst still demonstrated measurable activity, producing acetic acid at a rate of 0.24 mmol g_{cat}^{-1} h^{-1}. This confirms that the catalyst remains fundamentally active even without applied pressure, though the productivity is significantly lower than that under optimized pressurized conditions (18.2 mmol g_{cat}^{-1} h^{-1}). This difference is primarily attributed to the drastic reduction in the dissolved concentration of the gaseous reactants (CH_4 and CO) in the aqueous phase at ambient pressure, as governed by Henry's law. The inherently low concentration of reactants at the catalyst surface limits the reaction rate.

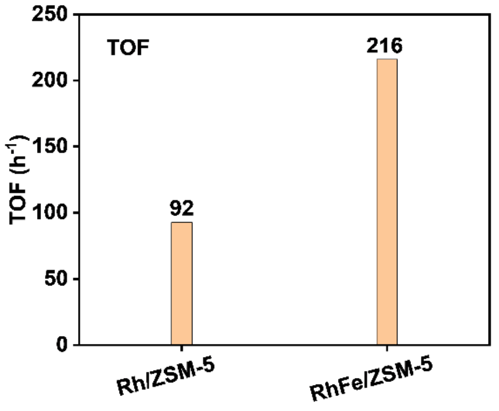

Supplementary Figure 5. TOF in Rh/ZSM-5 and RhFe/ZSM-5.

For the RhFe/ZSM-5 catalyst (0.55 wt% Rh, 0.25 wt% Fe), the TOF calculated based on total Rh + Fe atoms reached ~216 h^{-1}, which is ~2.5 times higher than that of monometallic Rh/ZSM-5 (~92 h^{-1}, calculated on total Rh). This demonstrates a clear enhancement in intrinsic activity per metal atom in the bimetallic system.

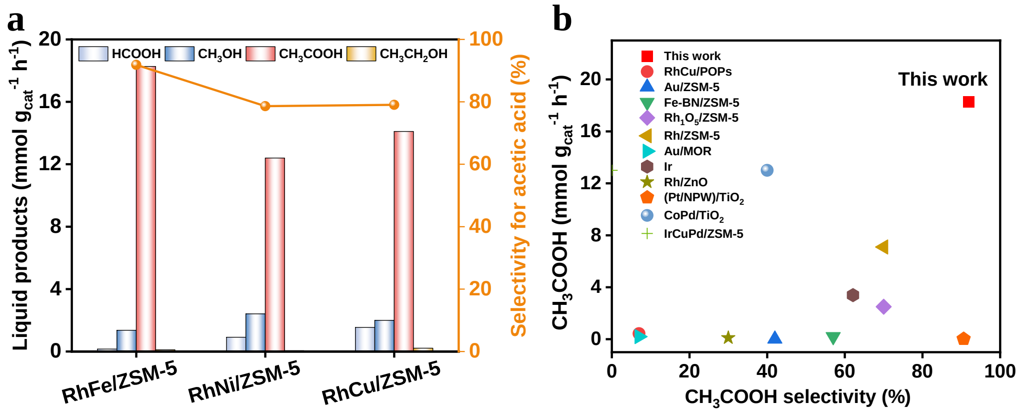

Supplementary Figure 6. (**a**) Catalytic performance over RhFe/ZSM-5, RhNi/ZSM-5, and RhCu/ZSM-5, reaction conditions: 463 K, 10 mg catalyst, 2 h, 20 mL H_2O, 40 bar CH_4, 3 bar O_2, 6 bar CO. (**b**) The reported yields of acetic acid in direct oxidative carbonylation from methane.

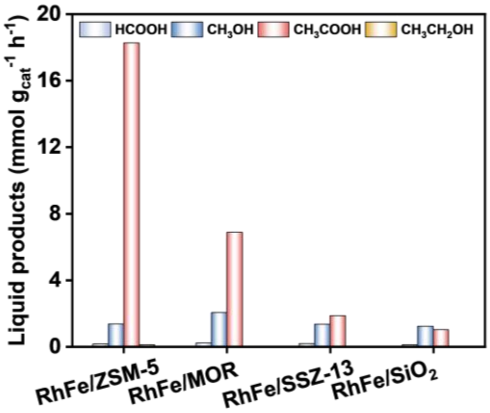

Supplementary Figure 7. Catalytic performance over different supports, reaction conditions: 463 K, 10 mg catalyst, 2 h, 20 mL H_2O, 40 bar CH_4, 3 bar O_2, 6 bar CO.

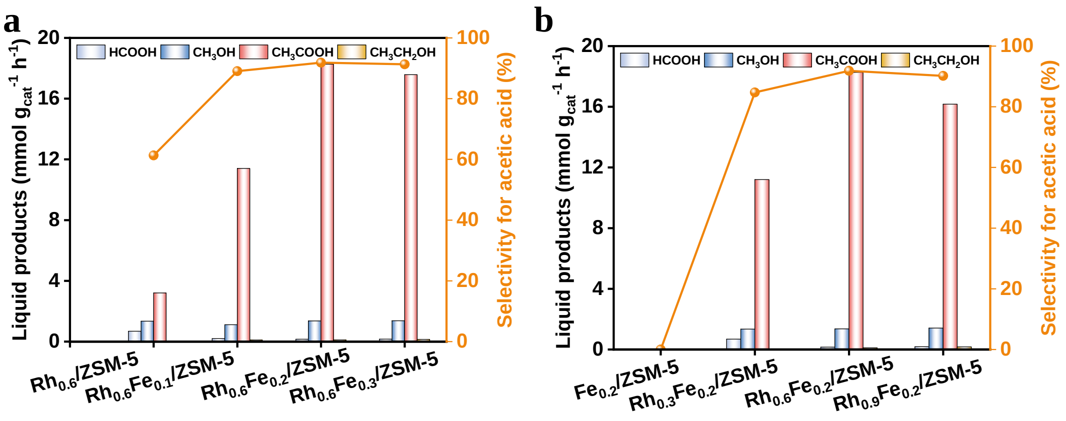

Supplementary Figure 8. (**a**) Catalytic performance of different Fe content. (**b**) Catalytic performance of different Rh content. Reaction conditions: 463 K, 10 mg catalyst, 2 h, 20 mL H_2O, 40 bar CH_4, 3 bar O_2, 6 bar CO.

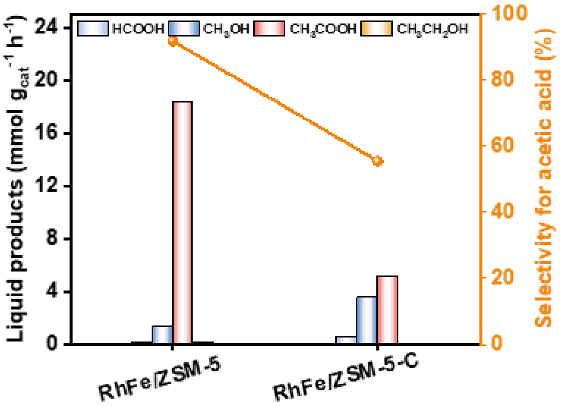

Supplementary Figure 9. Comparison of catalytic performance with commercial ZSM-5. Reaction conditions: 463 K, 10 mg catalyst, 2 h, 20 mL H_2O, 40 bar CH_4, 3 bar O_2, 6 bar CO.

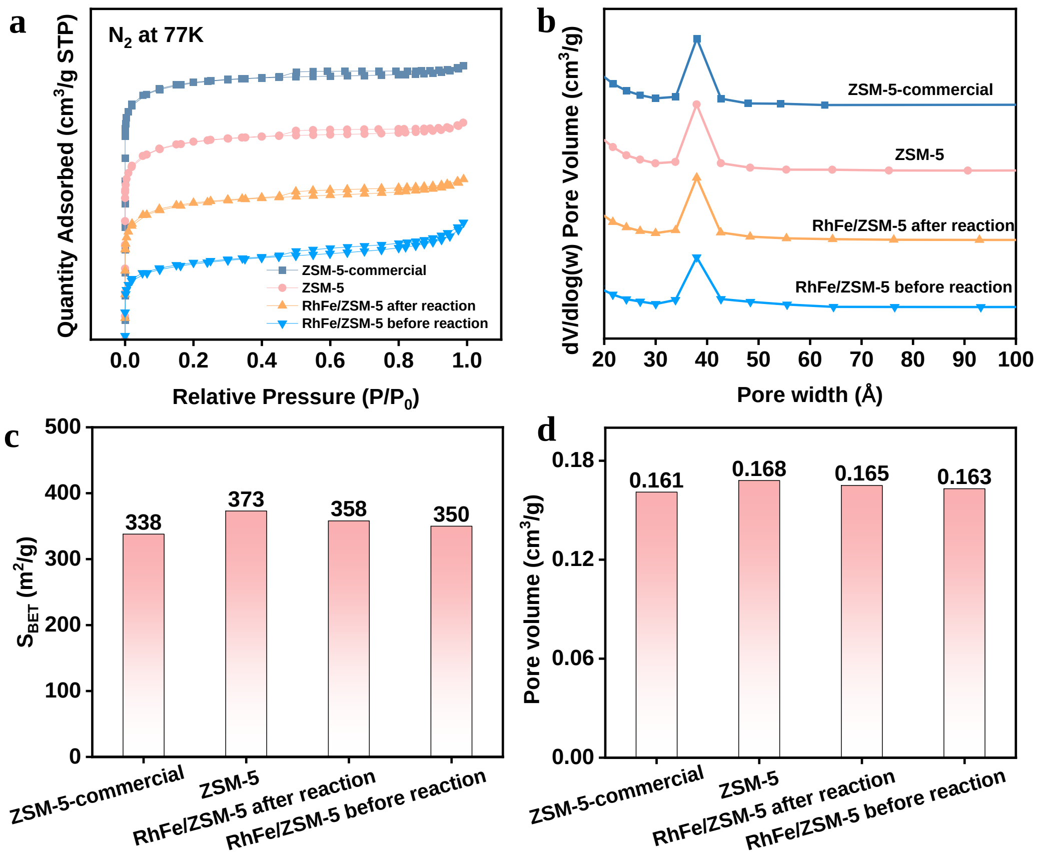

Supplementary Figure 10. The N_2 sorption measurements at 77 K of RhFe/ZSM-5 was synthesized using template-free seeded growth method and commercial ZSM-5**.** (**a**) N_2 adsorption-desorption isotherms at 77K. (**b**) The pore size distributions. (**c**) Surface area using BET method and (**d**) Pore volumes.

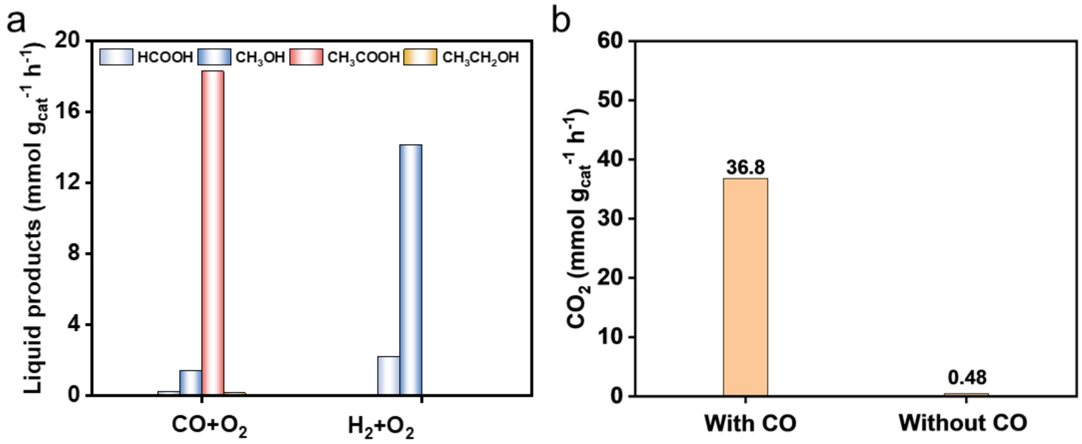

Supplementary Figure 11. (a) The yields of organic liquid products in CO + O_2, and H_2 + O_2. (**b**) The yields of CO_2 when feed acetic acid into the reaction in the presence and absence of CO, reaction condition: 463 K, 10 mg catalyst, 2 h, 20 mL H_2O, 3 bar O_2, 6 bar CO/or 6 bar Ar instead of CO, 0.175 mmol CH_3COOH.

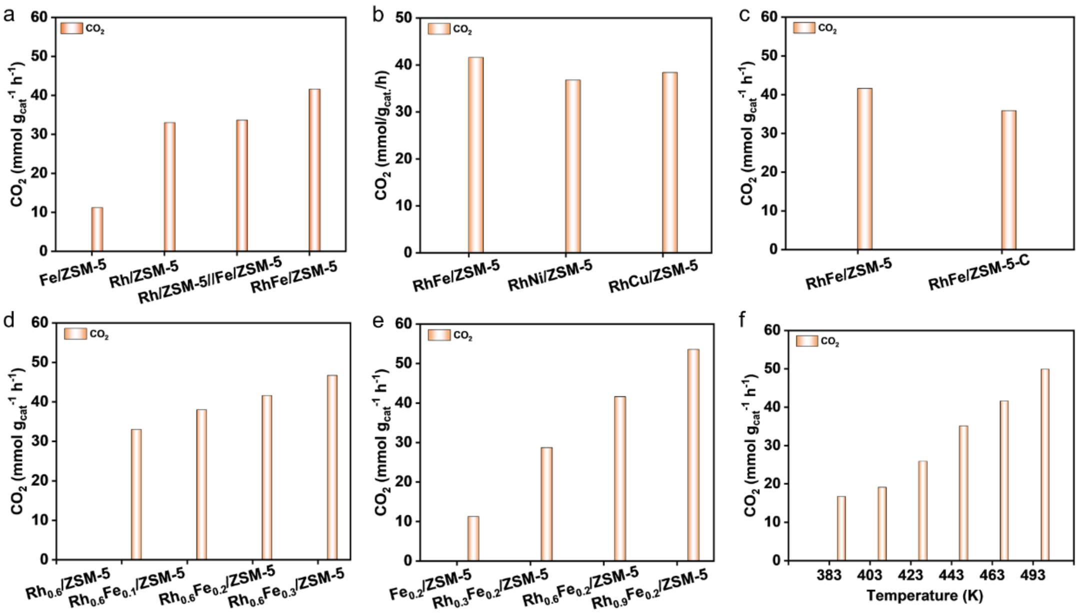

Supplementary Figure 12. (a-e) The content of CO_2 under different catalysts, reaction conditions, 10 mg catalyst, 3 h, 20 mL H_2O, 30 bar CH_4, 3 bar O_2, 6 bar CO. **(f)** The performance of CO_2 under different temperature.

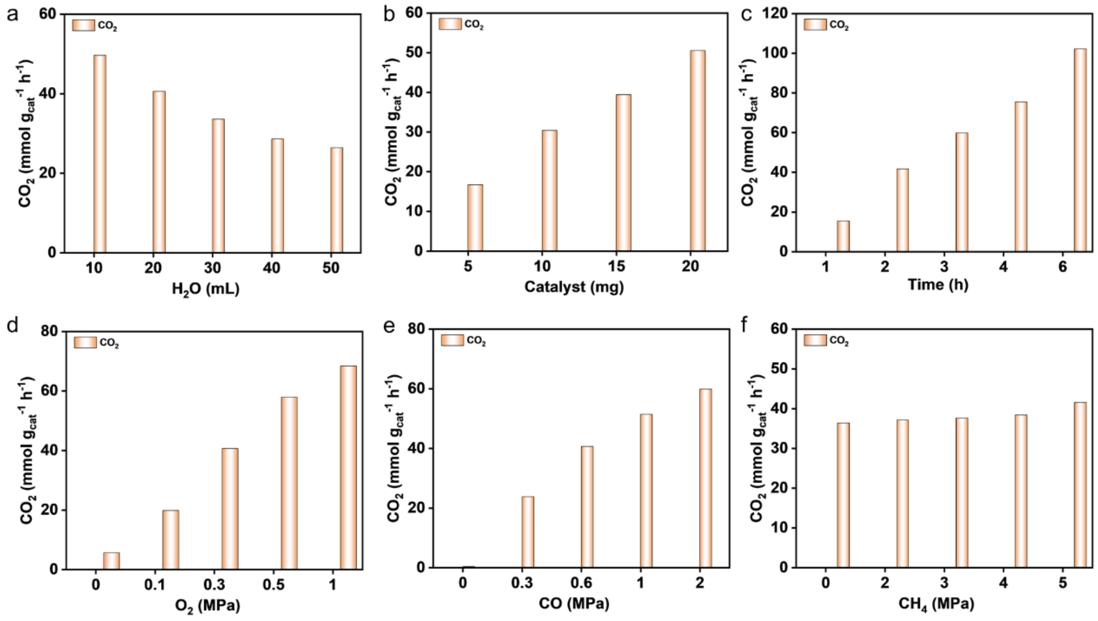

Supplementary Figure 13. Catalytic performance of CO_2. (**a**) Different H_2O volume; (**b**) Different amount of RhFe/ZSM-5 catalyst; (**c**) Different reaction time; (**d**) Different O_2 pressure; (**e**) Different CO pressure; (**f**) Different CH_4 pressure.

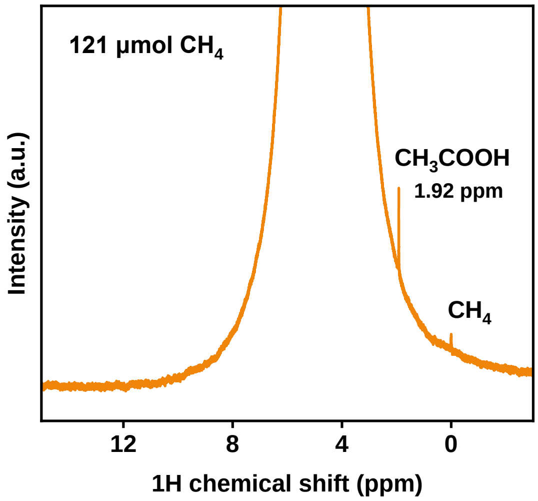

Supplementary Figure 14. ^{1}H NMR of CH_4 after reaction.

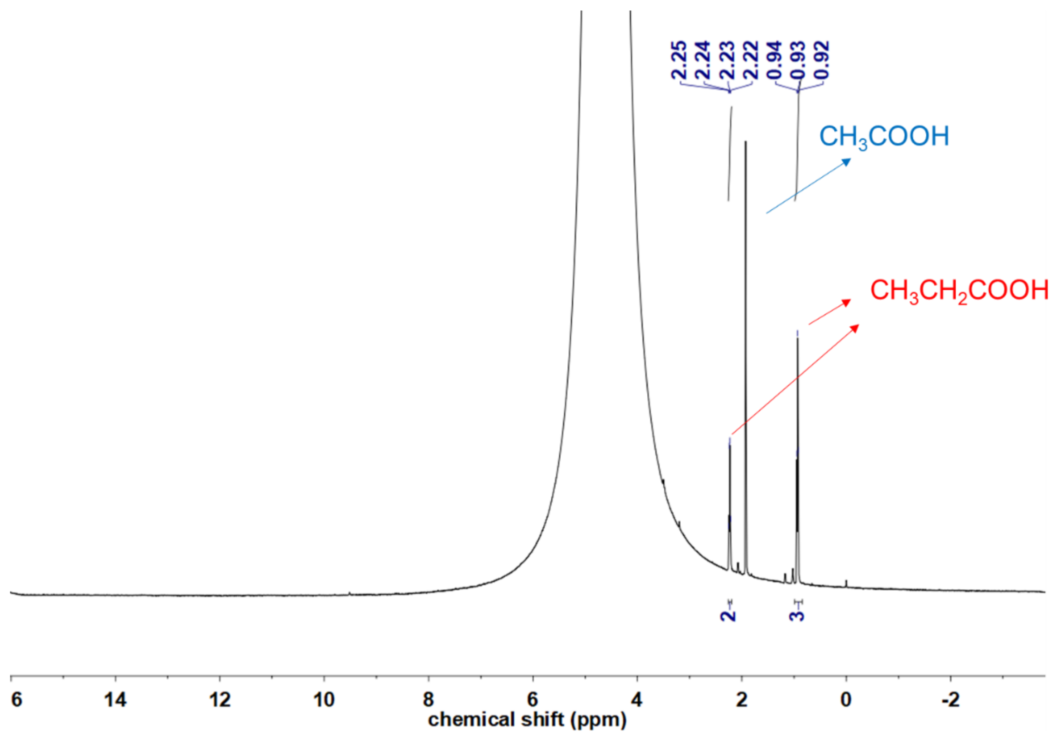

Supplementary Figure 15. ^{1}H NMR of CH_3CH_3 after reaction.

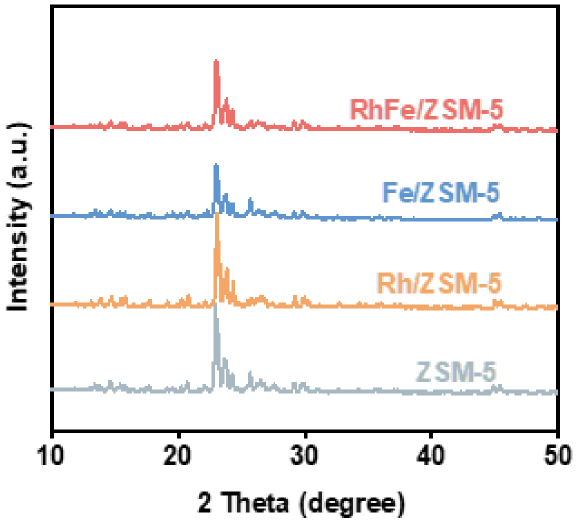

Supplementary Figure 16. XRD patterns of catalysts.

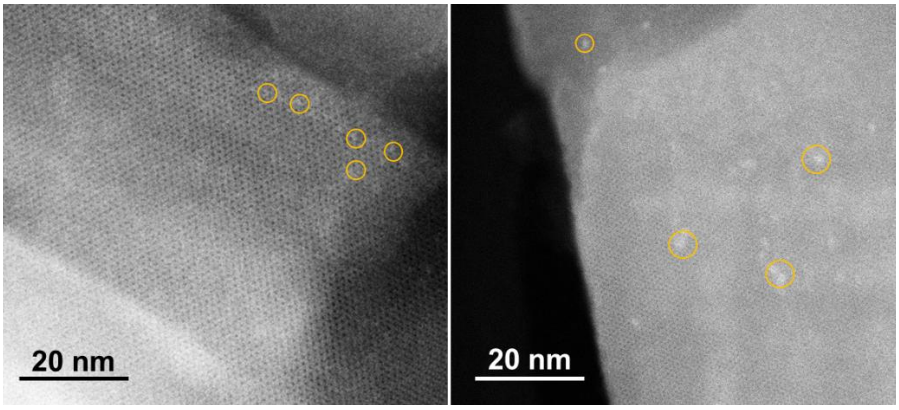

Supplementary Figure 17. AC-HAADF-STEM of RhFe/ZSM-5.

We performed detailed AC-HAADF-STEM analysis. High-resolution images (Supplementary Figure 17) clearly show the microporous structure of ZSM-5, dispersed nanoparticles, and single atoms distributed adjacently in multiple regions. By combining the results from CO-IR and EXAFS spectra (Supplementary Figure 24, Figure 2c-d), the existence of Rh nanoparticles and Fe single atoms has been demonstrated. These results directly reveal the spatial proximity between Rh and Fe, corroborating Mössbauer spectroscopy data, and providing visual morphological evidence for the Rh-O-Fe coordination structure (Figure 2e-f).

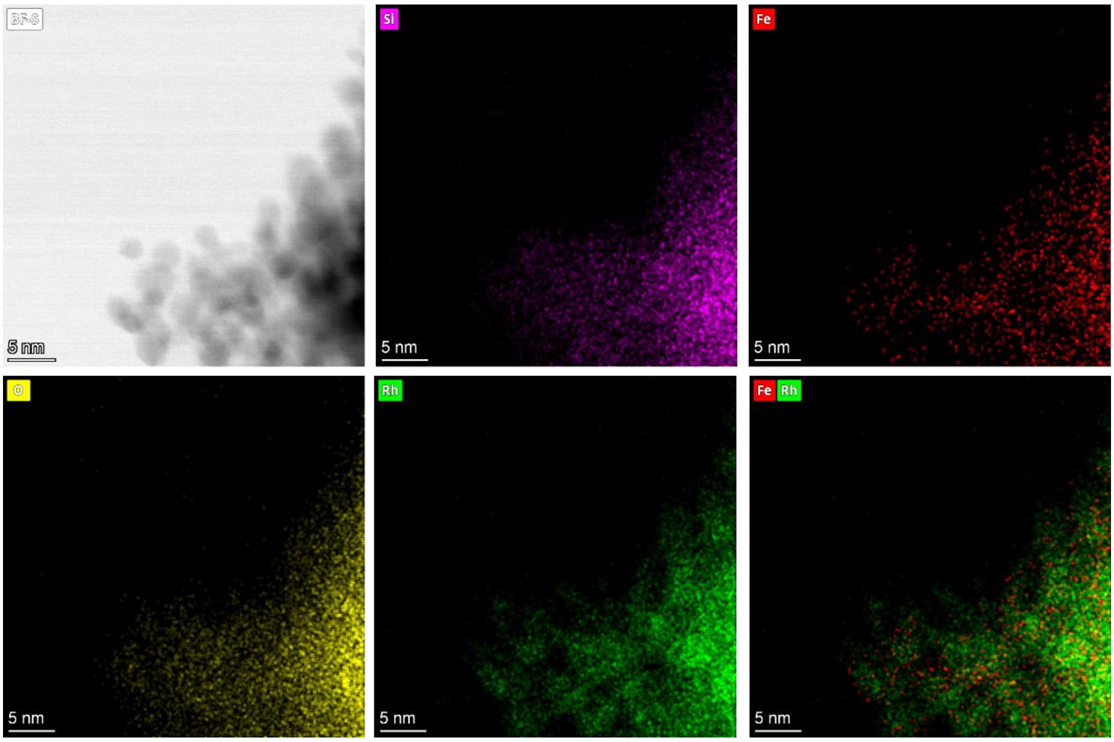

Supplementary Figure 18. Mapping image of RhFe/ZSM-5.

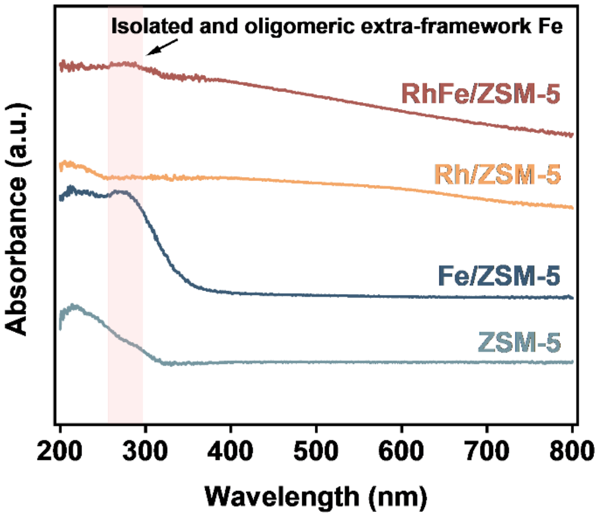

Supplementary Figure 19. UV-vis spectra for ZSM-5, Fe/ZSM-5, Rh/ZSM-5, and RhFe/ZSM-5.

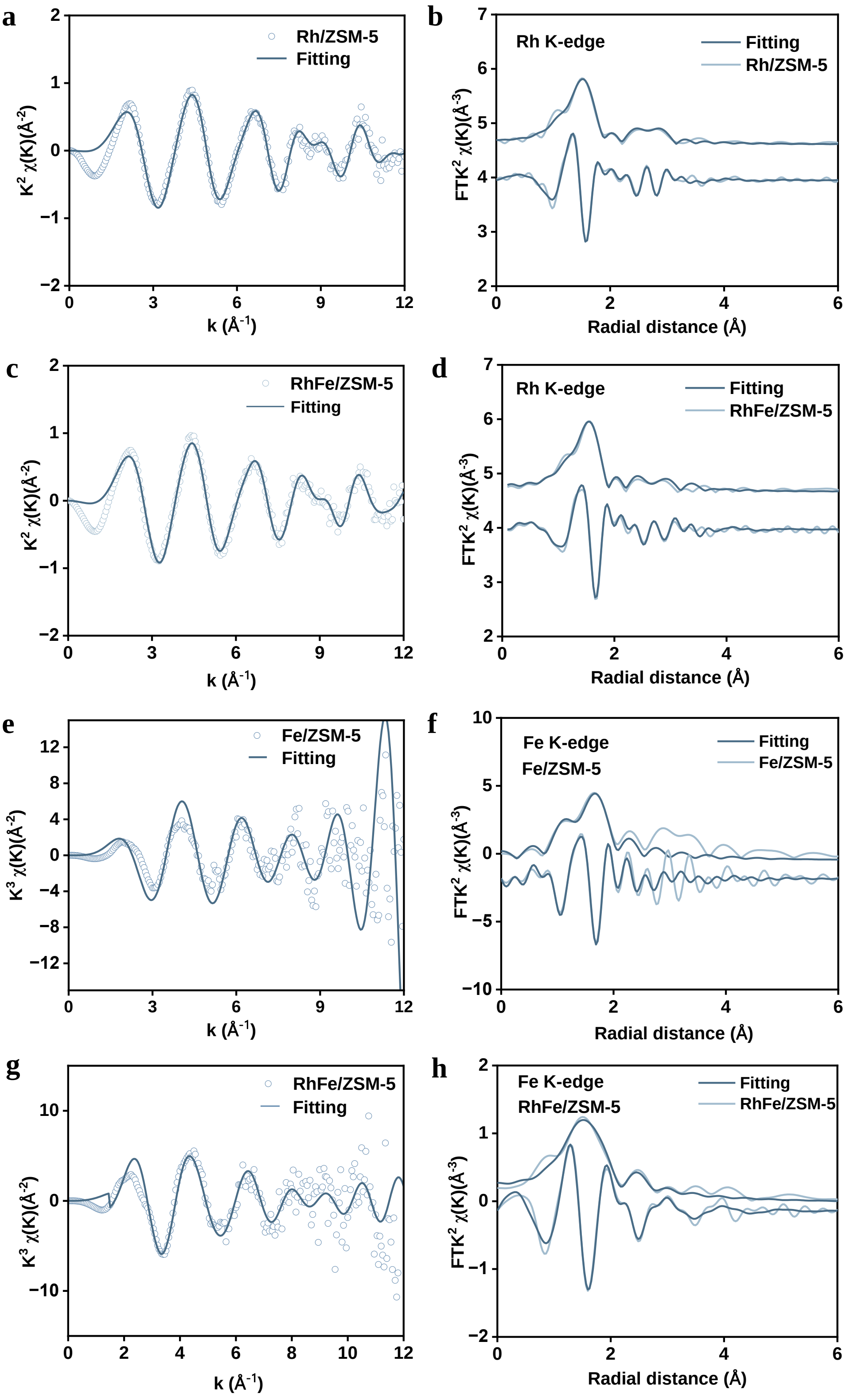

Supplementary Figure 20. **(a, c, e, g)** Fe and Rh K-edge EXAFS and curvefit analysis for Fe/ZSM-5, Rh/ZSM-5, and RhFe/ZSM-5. **(b, d, f, h)** Fe and Rh K-edge EXAFS and curvefit analysis for Fe/ZSM-5, Rh/ZSM-5, and RhFe/ZSM-5, shown in R-space (FT magnitude and imaginary component). The data not phase-corrected.

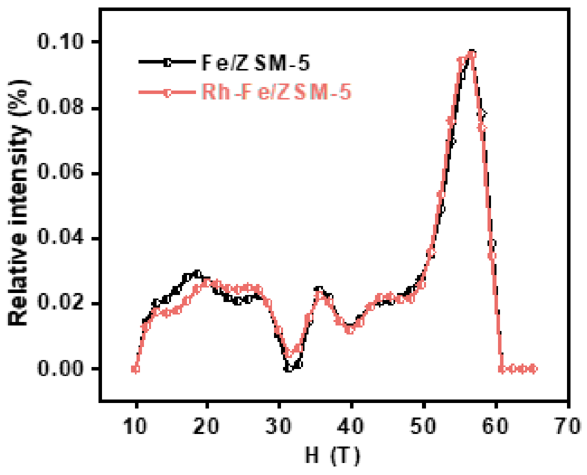

Supplementary Figure 21. The magnetic field distribution in ^{57}Fe Mössbauer spectra of Fe/ZSM-5 and FeRh/ZSM-5.

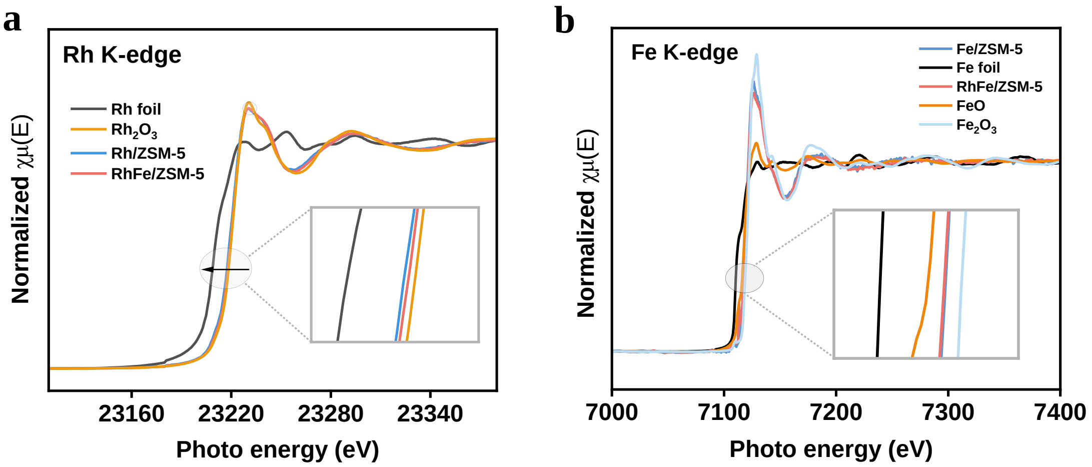

Supplementary Figure 22. (**a**) XANES spectra at the Rh K-edge of the Rh/ZSM-5, FeRh/ZSM-5, Rh_2O_3 and Rh foil. (**b**) XANES spectra at the Fe K-edge of the Fe/ZSM-5, FeRh/ZSM-5, FeO, Fe_2O_3 and Fe foil.

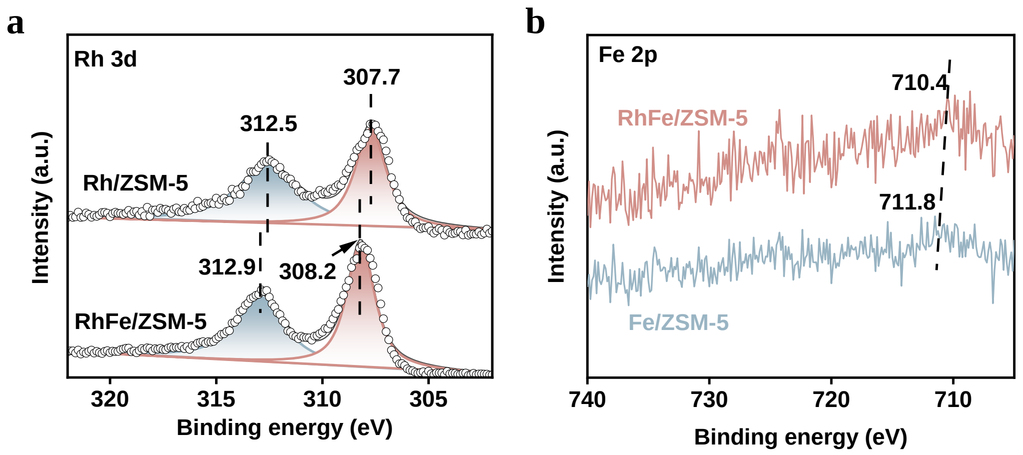

Supplementary Figure 23. Electron transfer. **(a)** Rh 3*d* XPS spectra of Rh/ZSM-5 and FeRh/ZSM-5. (**b**) Fe 2*p* XPS spectra of Fe/ZSM-5 and FeRh/ZSM-5, respectively.

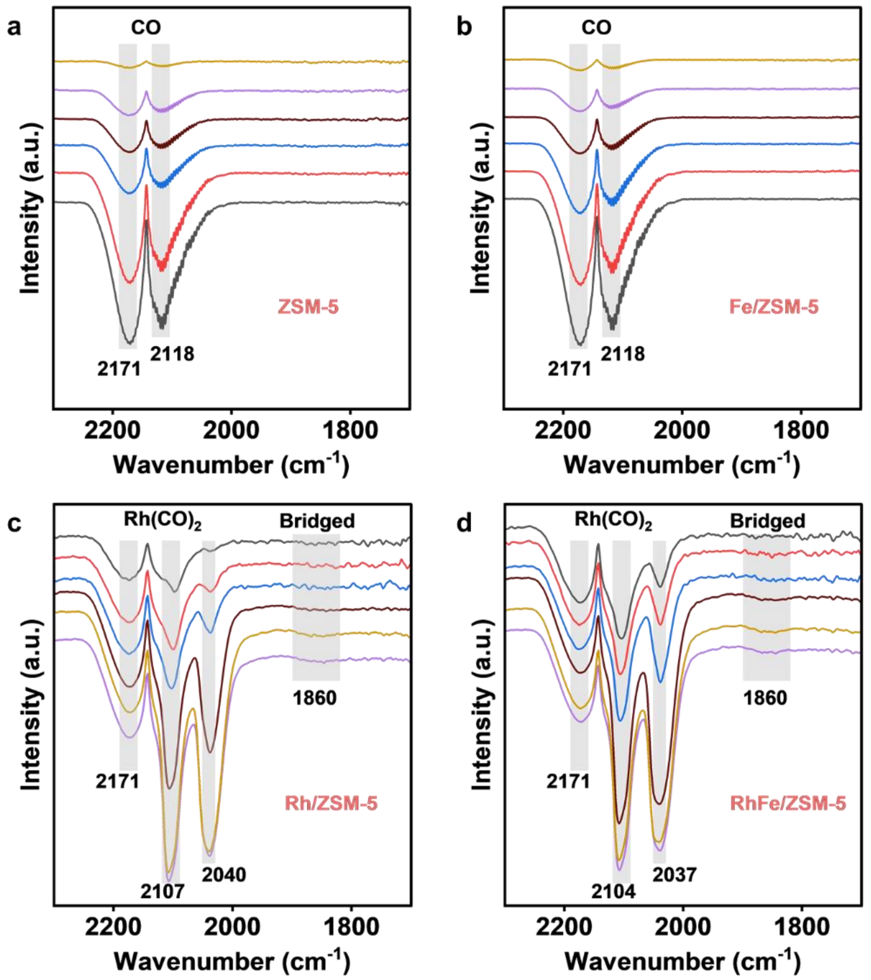

Supplementary Figure 24. CO-IR adsorption spectrum.

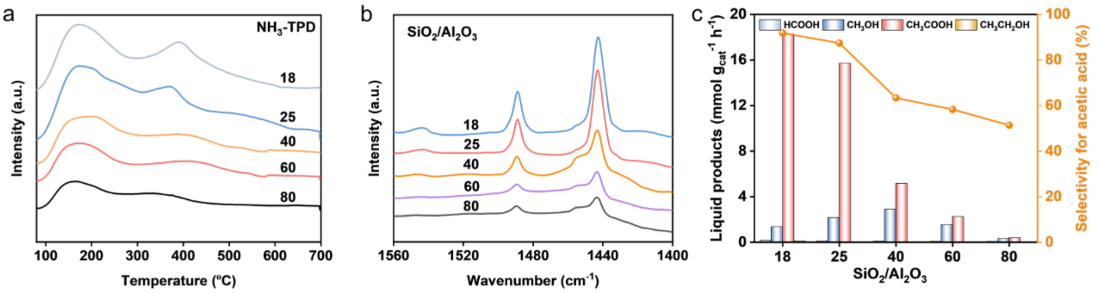

Supplementary Figure 25. (**a**) NH_3-TPD with different SiO_2/Al_2O_3 ZSM-5 catalysts. (**b**) Infrared spectra of different SiO_2/Al_2O_3 catalysts. (**c**) The performance of different SiO_2/Al_2O_3 catalysts.

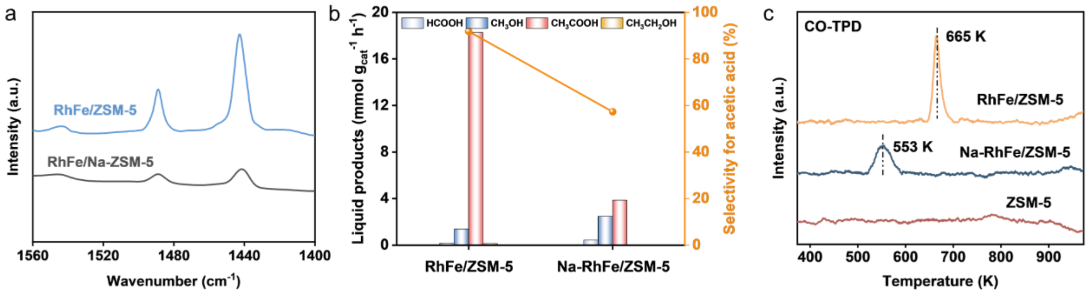

Supplementary Figure 26. (a) Infrared spectra of pyridine for RhFe/ZSM-5 and Na-RhFe/ZSM-5 catalysts. (**b**) The performance of RhFe/ZSM-5 and Na-RhFe/ZSM-5 catalysts. (**c**) CO-TPD spectra.

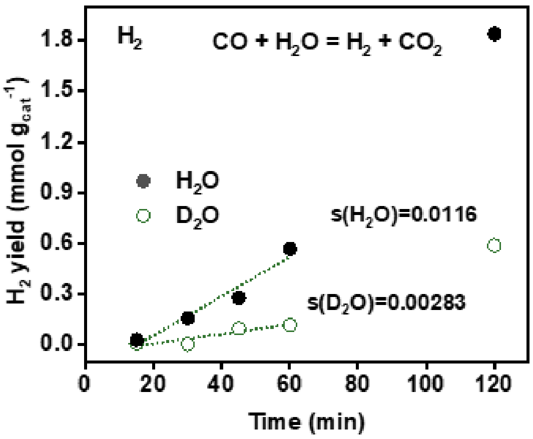

Supplementary Figure 27. H_2 production in H_2O and D_2O with CO at different time over RhFe/ZSM-5.

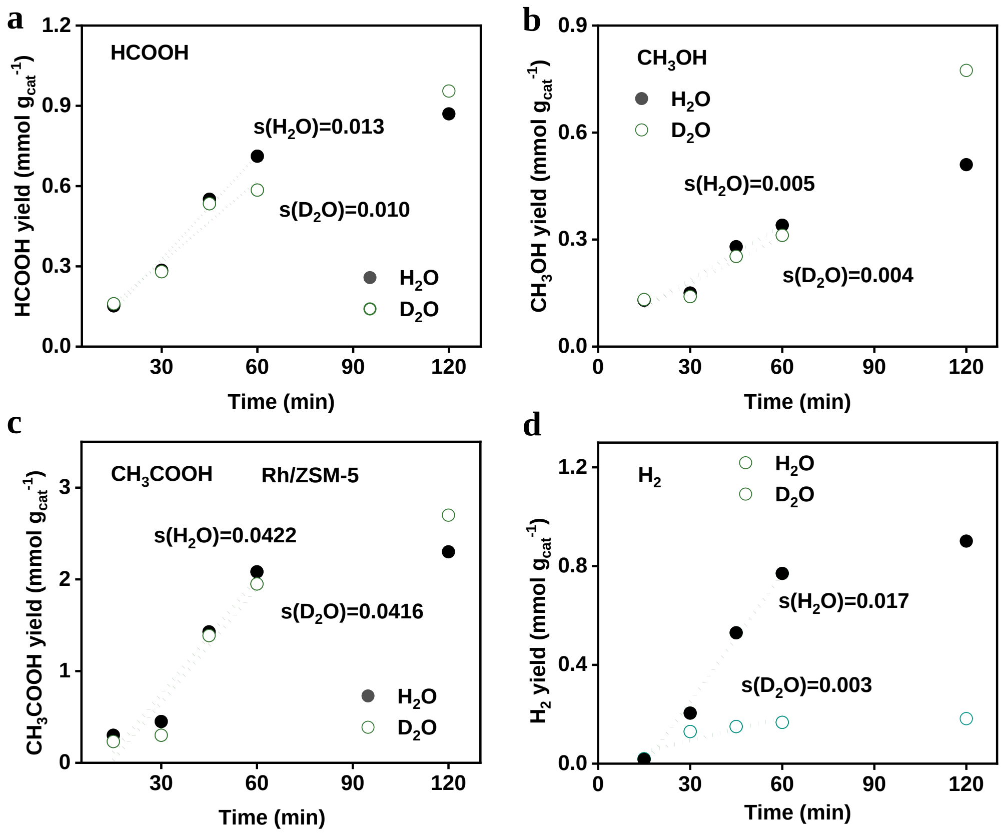

Supplementary Figure 28. Isotope effect ratio (KIE) for H_2O/D_2O with different time in Rh/ZSM-5.

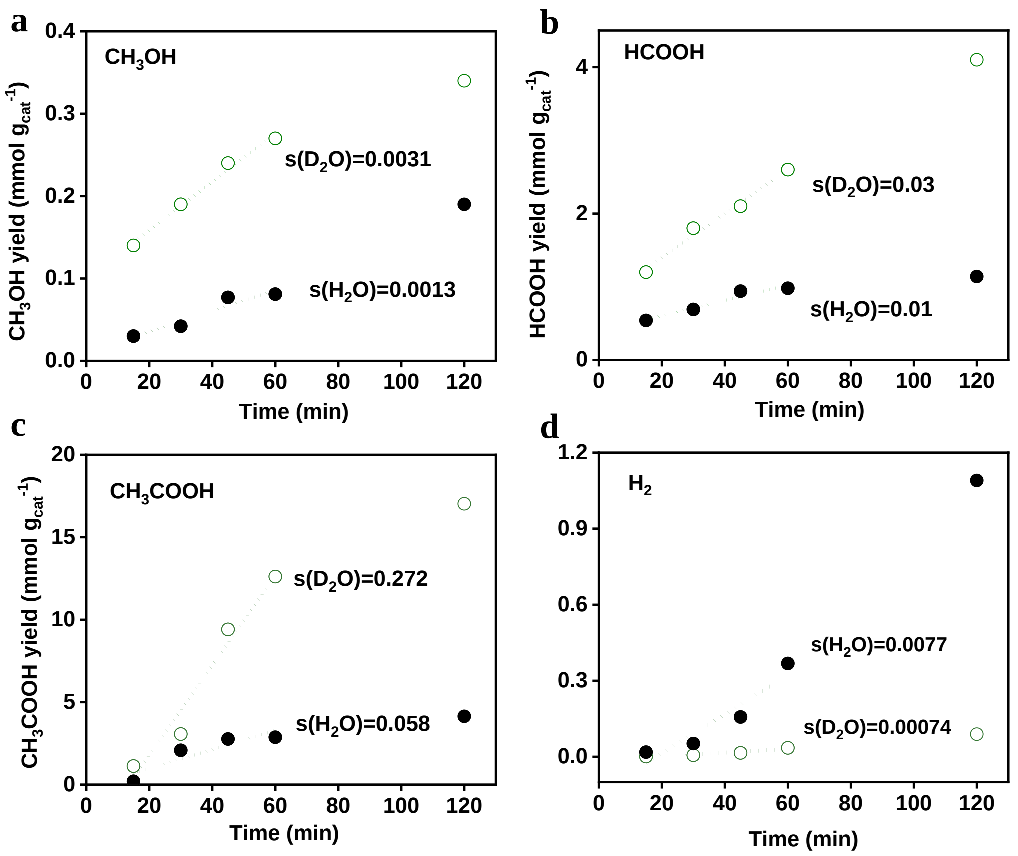

Supplementary Figure 29. Isotope effect ratio (KIE) for H_2O/D_2O with different time in RhFe/ZSM-5.

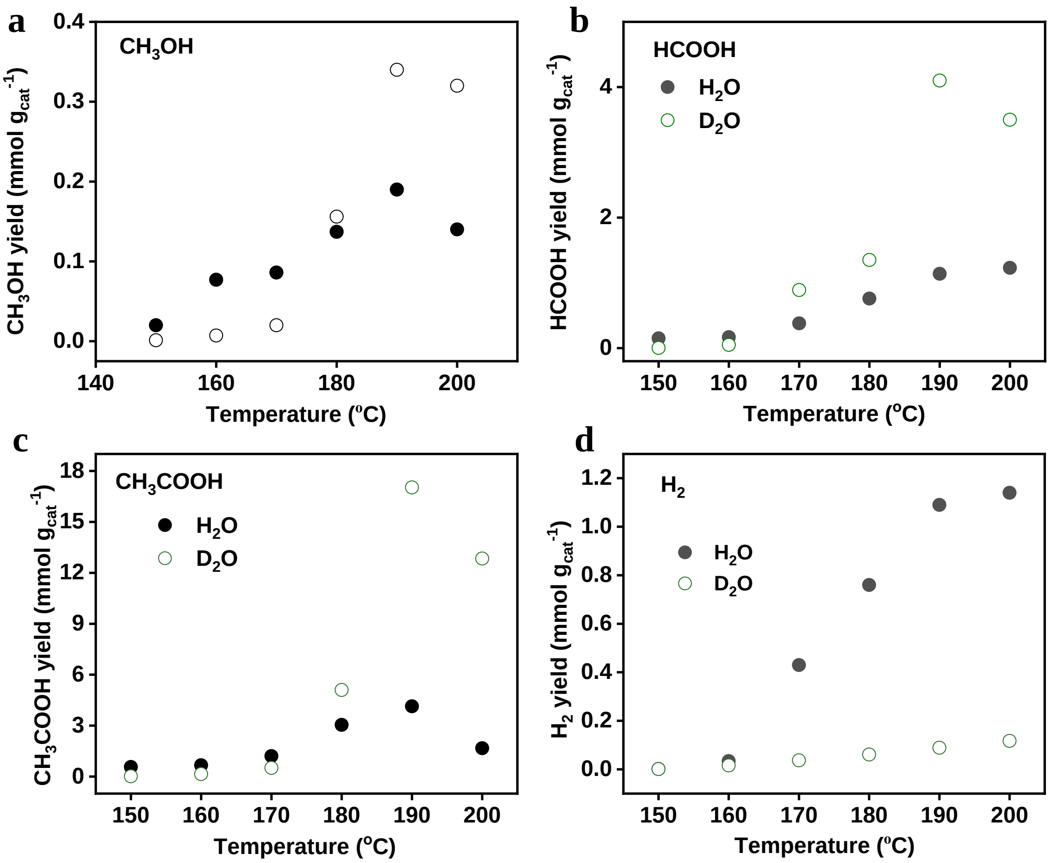

Supplementary Figure 30. Isotope effect ratio (KIE) for H_2O/D_2O with different temperature in RhFe/ZSM-5.

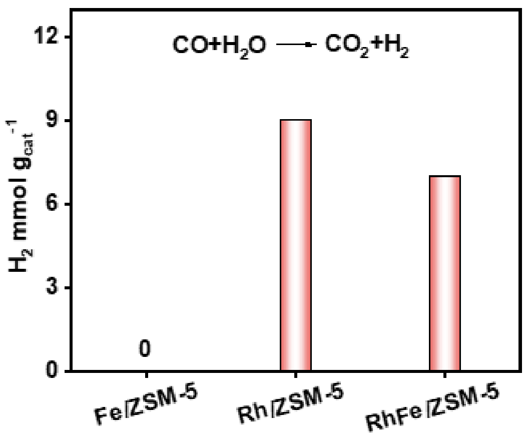

Supplementary Figure 31. H_2 production in water-gas shift reaction. Reaction conditions: 20 mL H_2O, 0.6 MPa CO, 463 K, 3 h.

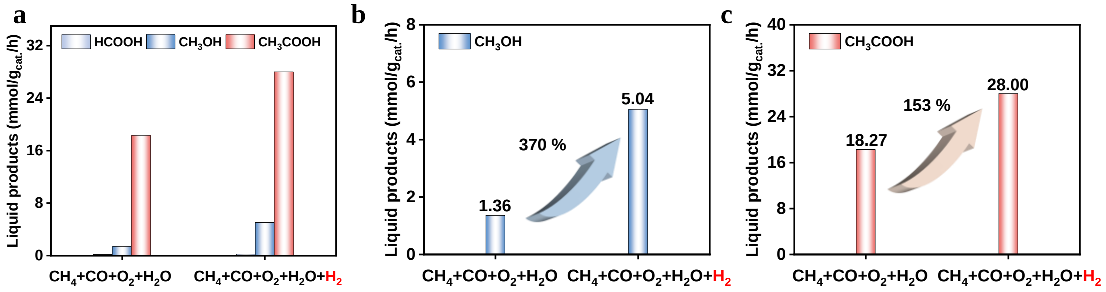

Supplementary Figure 32. The effect of additional H_2.

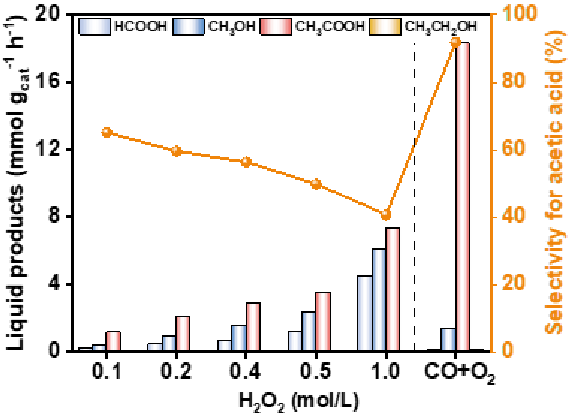

Supplementary Figure 33. The comparison of H_2O_2 with CO and O_2. Reaction conditions: 463 K, 10 mg catalyst, 2 h, 20 mL H_2O, 30 bar CH_4, 3 bar O_2, 3 bar Ar as balance gas, 6 bar CO.

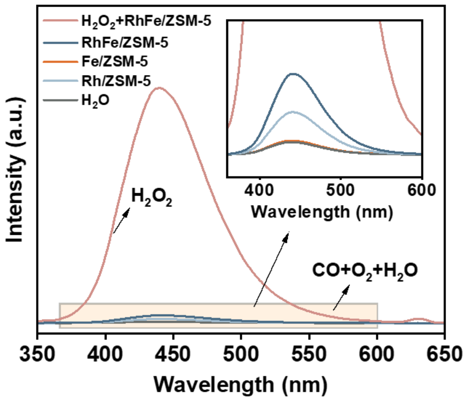

Supplementary Figure 34. The PL spectra of 2-hydroxyterephthalic acid using terephthalic acid as the probe molecule under O_2 + CO and H_2O_2.

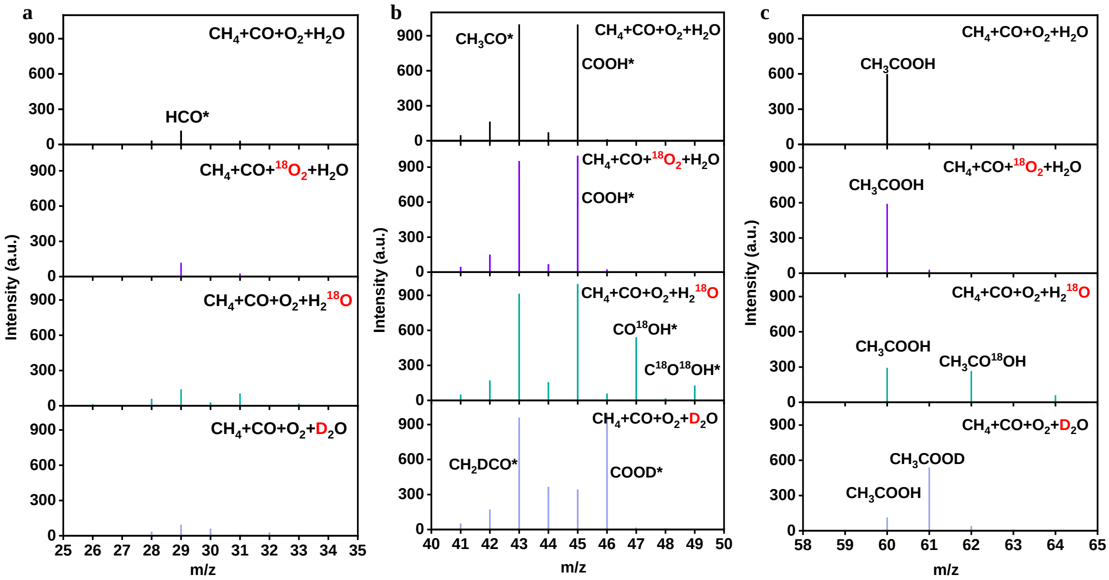

Supplementary Figure 35. GC-MS spectra of the isotope CH_3COOH produced from CH_4 conversion when using CH_4+CO+$^{16}O_2$+$H_2{}^{18}O$, CH_4+CO+$^{18}O_2$+$H_2{}^{16}O$ and CH_4+CO+O_2+D_2O as the reactants. (**a**) m/z from 25 to 35. (**b**) m/z from 40 to 50. (**c**) m/z from 58 to 65.

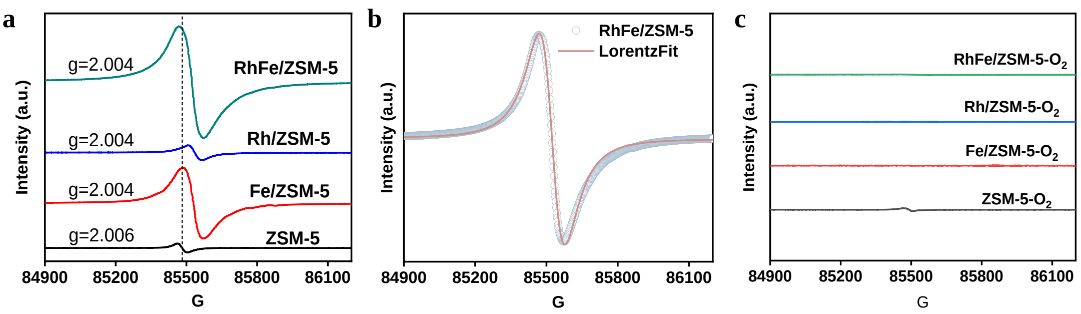

Supplementary Figure 36. (**a**) High-field EPR of fresh catalyst, (**b**) Lorentz fitting of RhFe/ZSM-5 catalyst. (**c**) After O_2 treatment at 463 K. (Test conditions: 15 K, microwave frequency: 240 Hz)

From the Mössbauer spectra, we can observe that Fe can be oxidized to high spin $Fe^{(IV)}$. Although the valence state change of Rh is not directly visible, the significant alteration in the fine structure of the RhFe catalyst after Rh incorporation suggests that the valence state of Rh may also undergo considerable changes.

To better investigate the role of Rh and Fe during the reaction, we further employed high-frequency (240 Hz) quasi in-situ electron paramagnetic resonance (HF-EPR) spectroscopy to probe the valence state changes of the metals sites.

Before the reaction, signals were detected for ZSM, Fe/ZSM, Rh/ZSM, and RhFe/ZSM (Supplementary Figure 27a). A weak narrow signal (g = 2.006) appeared in ZSM, likely due to defect sites in the ZSM-5 support. After metal loading, the g-values of Fe/ZSM and Rh/ZSM shifted to 2.004 with an increased peak width, characteristic of metal site signals. The RhFe/ZSM catalyst exhibited a stronger metal signal, and the fitting analysis revealed only a single set of peaks (Supplementary Figure 27b), indicating a magnetic exchange interaction among Fe, Rh, and ZSM. This further confirms that Fe and Rh are in close proximity with strong interactions. Combined with XPS and XAS analysis, both Fe and Rh were found to be in a mixed valence state between +2 and +3. Therefore, the observed EPR signal corresponds to $Fe^{(III)}$ and $Rh^{(II)}$ in this system.

After O_2 activation at 463 K, the HF-EPR signal for Fe/ZSM-5 disappeared (Supplementary Figure 27c), suggesting that Fe was fully oxidized to $Fe^{(IV)}$ (S=2) under an oxygen atmosphere. The loss EPR signal of $Fe^{(IV)}$ can be attributed to its high-spin

(S=2) configuration, where zero-field splitting (ZFS) causes significant broadening of the signal, effectively quenching it under the measurement conditions. The simultaneous disappearance of the HF-EPR signals for both Fe/ZSM-5 and FeRh/ZSM-5 indicates that, along the oxidation of $Fe^{(III)}$ (S=5/2) to $Fe^{(IV)}$ (S=2), $Rh^{(II)}$ (S=1/2) was oxidized to $Rh^{(III)}$ (S=0).

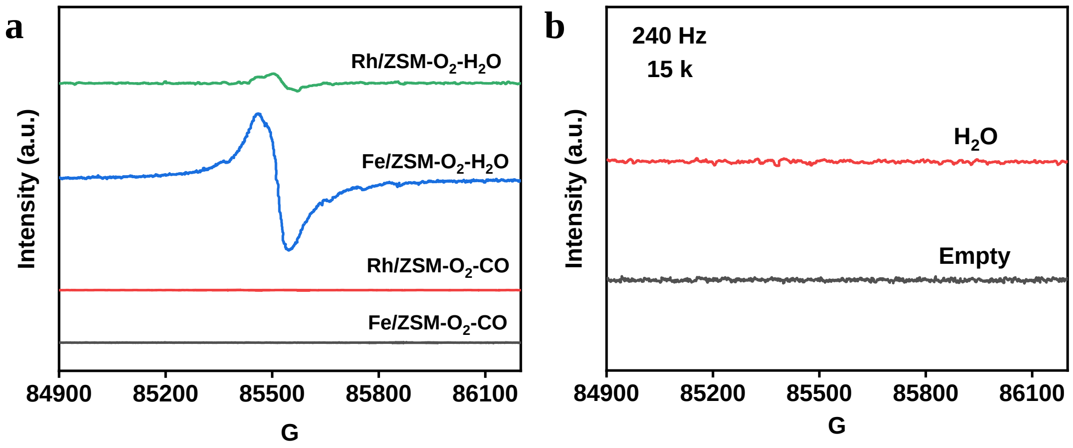

Supplementary Figure 37. (**a**) High-field EPR spectra of an empty tube and pure water, (**b**) Effects of CO and H_2O on Fe/ZSM-5 and Rh/ZSM-5 catalysts. Reaction conditions: 463 K, 12 h, test conditions: 15 K, microwave frequency: 240 Hz.

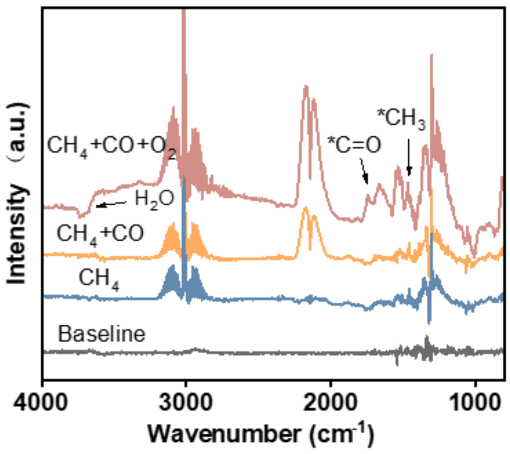

Supplementary Figure 38. In-situ DRIFTS spectra at CH_4, O_2, CO, and H_2O at 463 K.

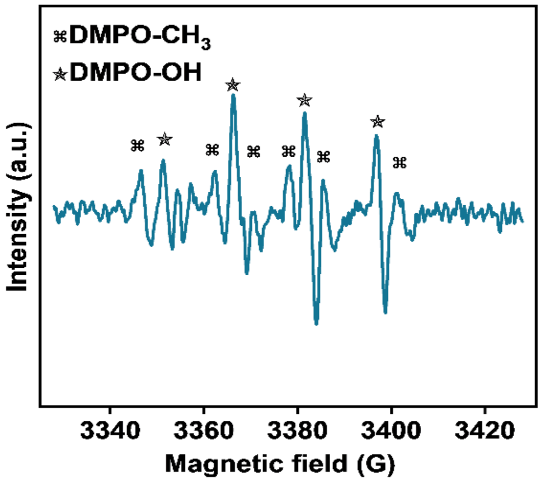

Supplementary Figure 39. In-situ EPR spectra under CH_4+CO+O_2+H_2O.

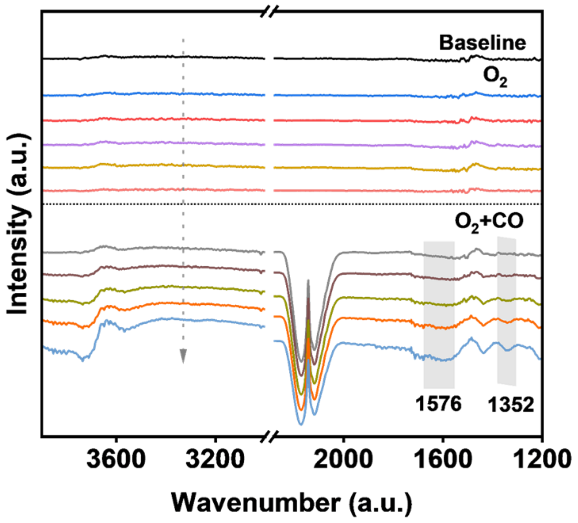

Supplementary Figure 40. In-situ diffuse reflectance infrared fourier transform spectra collected at the H_2O +O_2 and O_2/H_2O/CO at 463 K over Fe/ZSM-5.

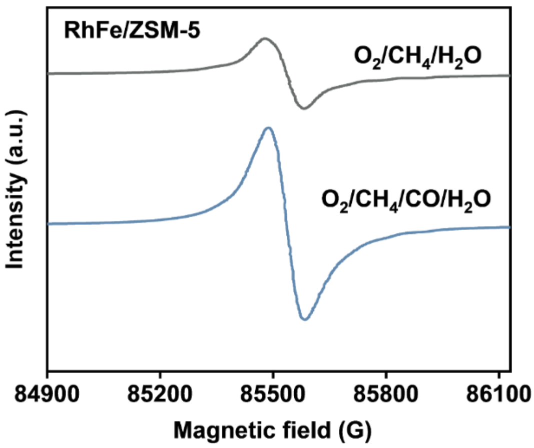

Supplementary Figure 41. High-field EPR spectra of RhFe/ZSM-5 in $O_2/CH_4/H_2O$ and $O_2/CH_4/CO/H_2O$. Reaction conditions: 463 K, 12 h, test conditions: 15 K, microwave frequency: 240 Hz.

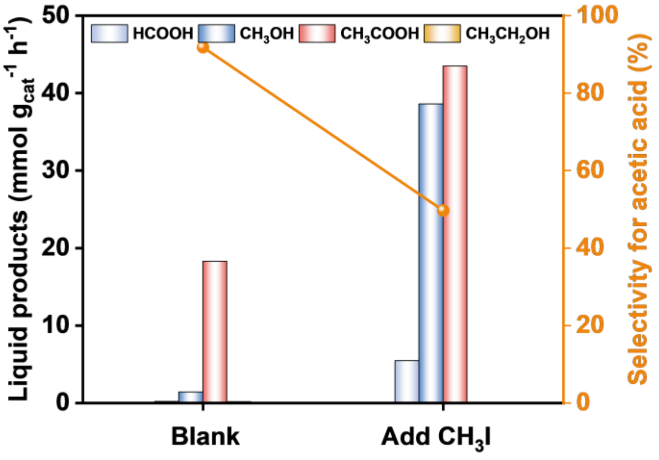

Supplementary Figure 42. The liquid products after adding CH_3I.

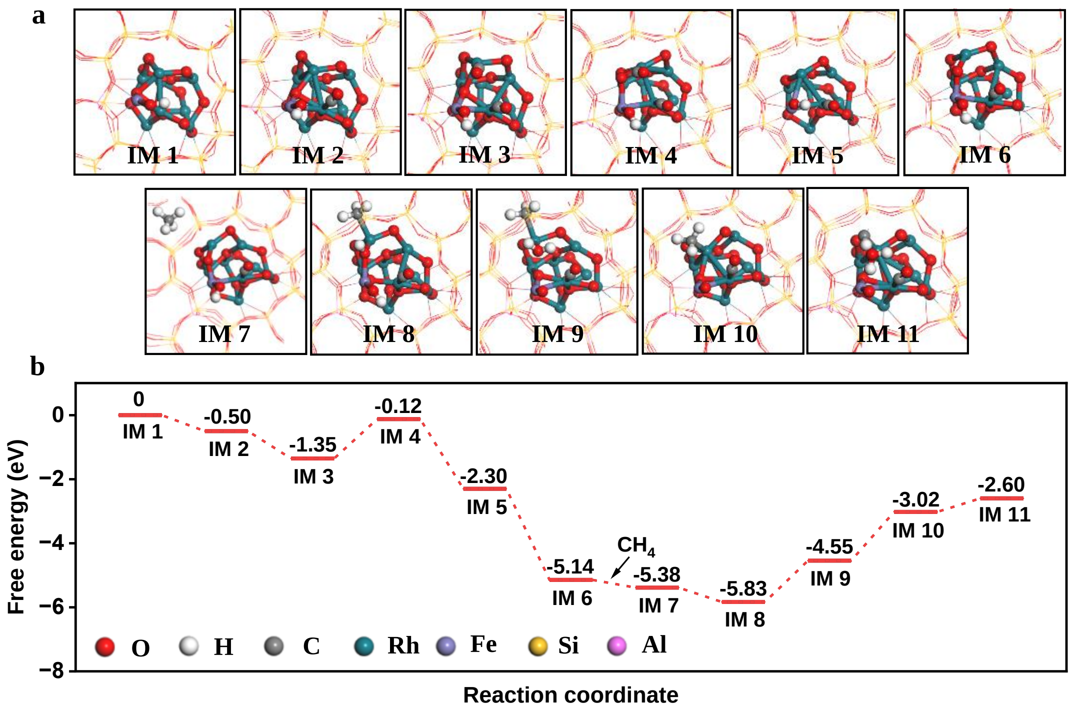

Supplementary Figure 43. DFT calculations. (**a**) Models of different reaction intermediate. (**b**) Energy profiles for the conversion of $CO/O_2/CH_4/H_2O$.

The overall reaction mechanism is supported by both experimental observations and DFT calculations. The strong coordination affinity of carbon monoxide to rhodium facilitates the sequential adsorption of two CO molecules onto Rh nanoparticles, releasing adsorption energies of 0.50 eV and 0.85 eV, respectively. One of the adsorbed CO species undergoes oxidation to CO_2 via a bridging oxygen atom within the Rh-O-Fe interface, generating an oxygen vacancy that subsequently activates molecular O_2. Methane shows weak adsorption on Rh sites (ΔG_{ads} = -0.24 eV), consistent with its intrinsically low surface affinity. The activated oxygen species could oxidize Rh sites, which could promote the C-H bond activation of methane. Simultaneously, these oxygen species could oxidize surface Fe-OH to form high-valent $Fe^{(IV)}$=O species, which in turn activate water molecules and generate •OH radicals. The resulting •OH radicals readily couple with adjacent CO adsorbed on Rh sites to form *COOH intermediates. DFT calculations show that the subsequent coupling between $*CH_3$ and *COOH to yield CH_3COOH proceeds with a low energy barrier of 0.48 eV, highlighting the thermodynamic and kinetic favorability of this pathway.

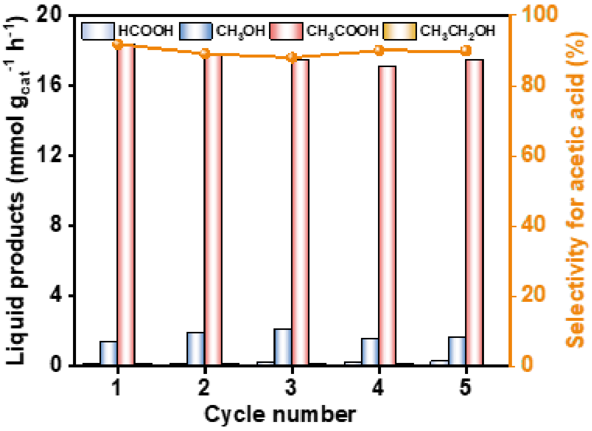

Supplementary Figure 44. Cycle number of ReFe/ZSM-5 with five reactions. Reaction conditions: 463 K, 10 mg catalyst, 2 h, 20 mL H_2O, 40 bar CH_4, 3 bar O_2, 6 bar CO.

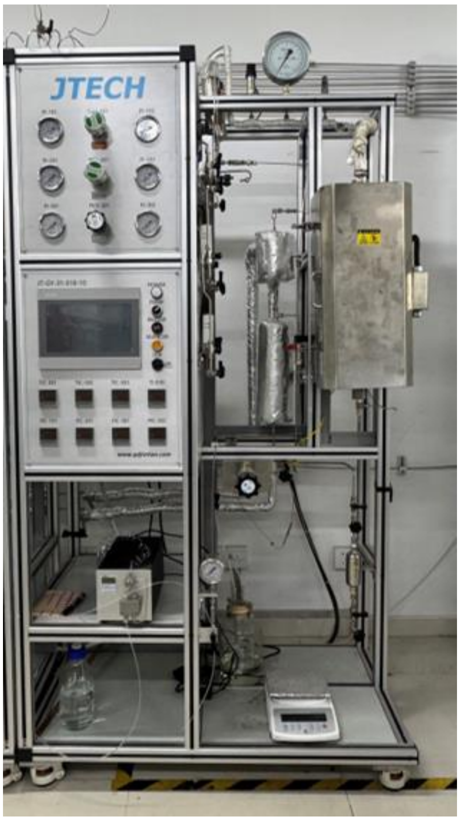

Supplementary Figure 45. The reactor used to evaluate catalytic performance of RhFe/ZSM-5 catalyst towards to the oxidative coupling of methane.

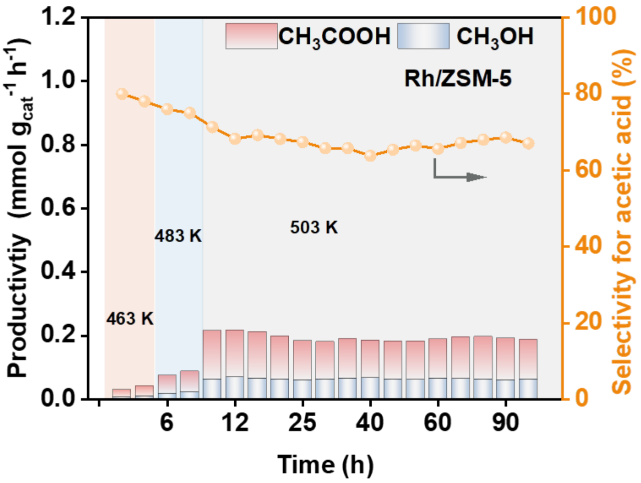

Supplementary Figure 46. The methane oxidative coupling reaction catalyzed in a continuous regime, reaction conditions: Prior to the reaction, 0.2 g of Rh/ZSM-5 catalyst (20-40 mesh) was thoroughly mixed with 0.6 g of acid-washed quartz sand (20-40 mesh) to minimize temperature gradients, and the mixture was loaded into a stainless-steel fixed-bed reactor. 503 K, H_2O 0.3 mL/min, CH_4 20 mL/min, CO 10 mL/min, O_2 5 mL/min.

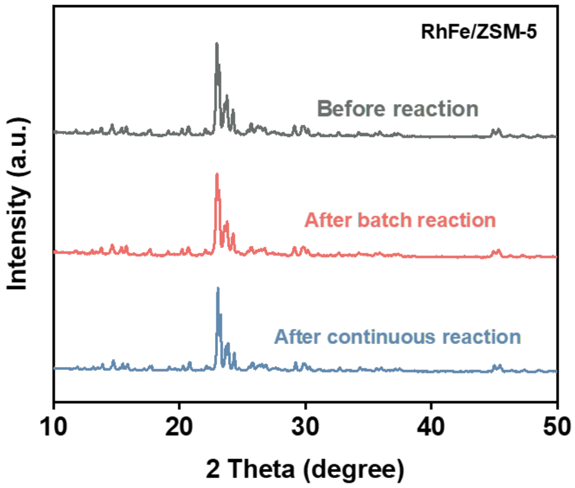

Supplementary Figure 47. XRD patterns of catalysts after batch and continuous reaction.

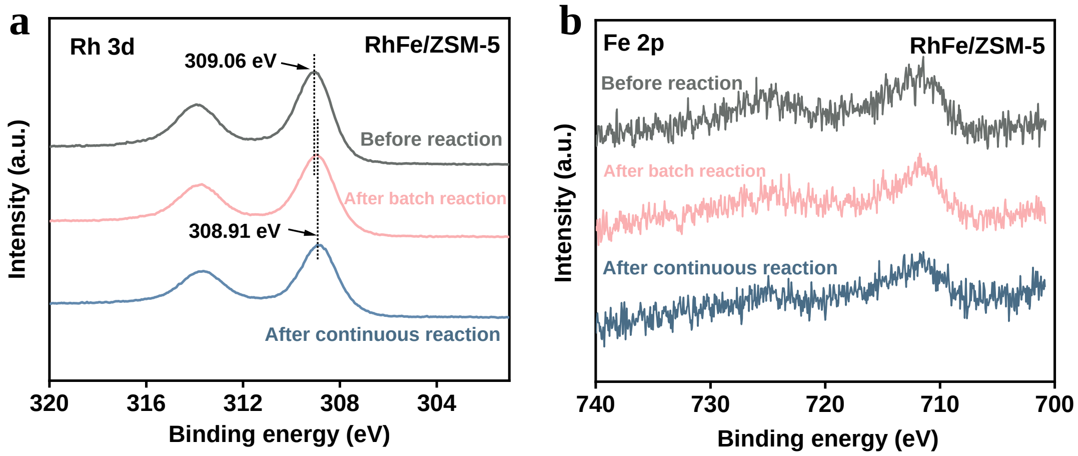

Supplementary Figure 48. (a) Rh 3d XPS spectra of RhFe/ZSM-5 after batch and continuous reaction. (**b**) Fe 2p XPS spectra of RhFe/ZSM-5 after batch and continuous reaction.

After batch and continuous reaction, the different binding energy of XPS indicated subtle electronic modifications of Rh species attributed to CO adsorption and π-backbonding.

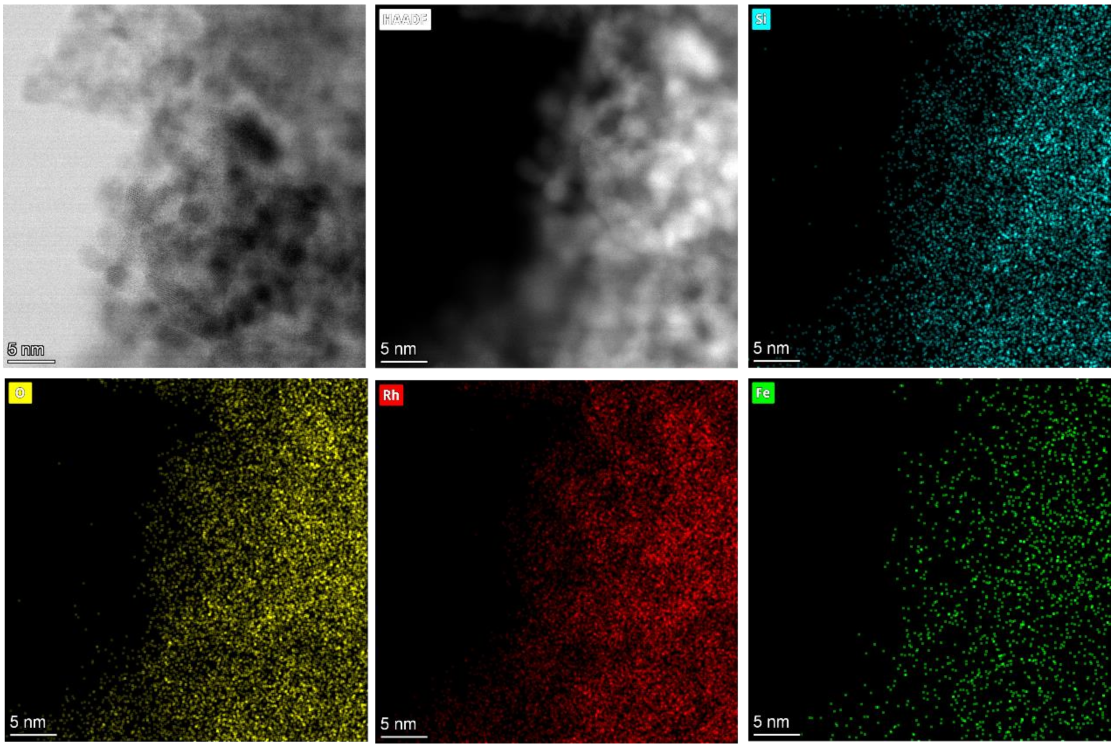

Supplementary Figure 49. Mapping image of RhFe/ZSM-5 after batch reaction.

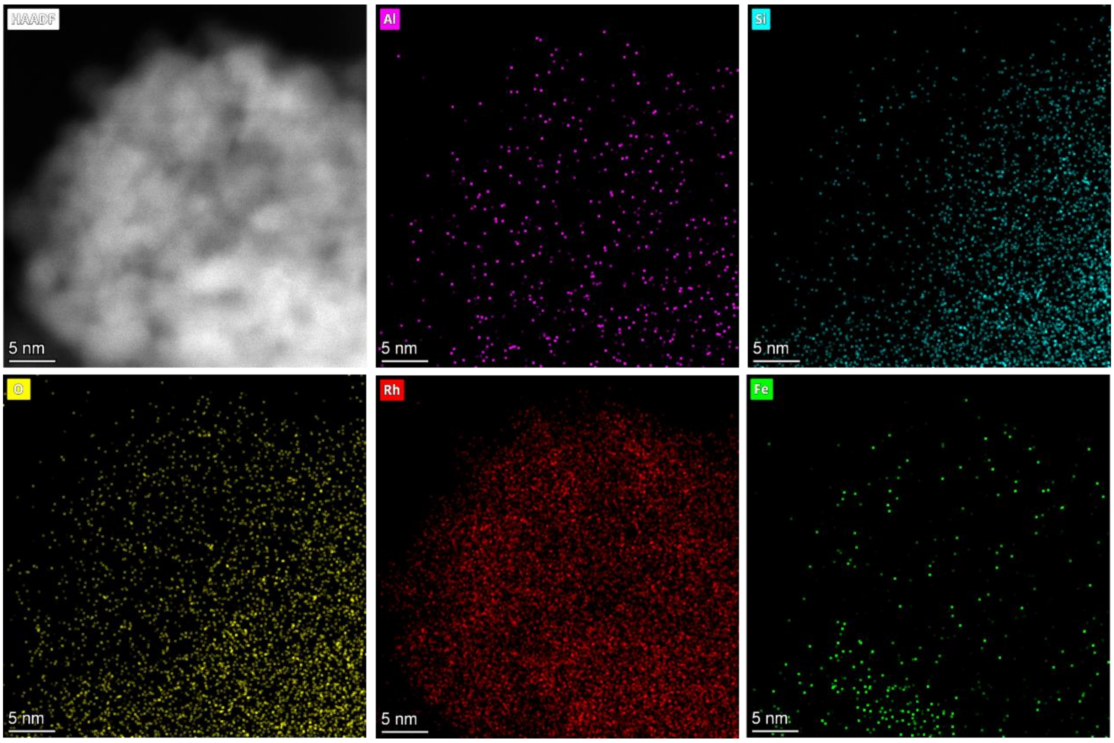

Supplementary Figure 50. Mapping image of RhFe/ZSM-5 after continuous reaction.

Supplementary Table 1. Comparisons with the representative catalytic performances on the yield and selectivity of CH_3COOH.

Entry	catalyst	Reaction condition	Time (h)	CH_3COOH Yield (mmol g_{cat}^{-1} h^{-1})	CH_3COOH Selectivity (%)	Ref.
1	FeRh/ZSM-5	10 mg catalyst, 50 bar CH_4, 3 bar O_2, 6 bar CO, 463 K, 20 mL H_2O	2	18.27	91.9	This Work
2	Rh_1-Cu/POPs	50 mg catalyst, 30 bar CH_4, 5 bar O_2, 10 bar CO, 423 K, 20 mL H_2O	2	0.43	7	1
3	Au/ZSM-5	100 mg catalyst, 20.7 bar CH_4, 3.5 bar O_2, 513 K, 15 mL H_2O	2	0.0051	42	2
4	Fe-BN/ZSM-5	30 mg catalyst, 25 bar CH_4, 654 umol H_2O_2, 5 bar CO, 293 K, 20 mL H_2O	6	0.2	57	3
5	Rh_1O_5/ZSM-5	28 mg, 50 bar CH_4, 8 bar O_2, 10 bar CO, 423 K, 10 mL H_2O	12	2.5	70	4
6	Rh/ZSM-5	20 mg, 20 bar CH_4, 5 bar O_2, 5 bar CO, 423 K, 20 mL H_2O	1	7.1	70	5
7	Au/MOR	100 mg, 20 bar CH_4, 5 bar O_2, 5 bar CO, 453 K, 20 mL H_2O	1	0.2	7	6
8	As-prepared Ir	15 mg, 19 bar CH_4, 4 bar O_2, 5 bar CO, 423 K, 15	3	3.39	62.1	7

		mL H_2O				
9	Rh/ZnO	R.T., 1.5 mg, 1 bar CH_4, 2.2 V vs RHE.	Continuous	0.105	30	8
10	Pd^{2+}	3 mL 96% H_2SO_4, 453 K, 20mM $PdSO_4$; 400 psi CH_4; 150 psi O_2	4	284mM	71	9
11	(Pt/NPW)/TiO_2	50 mg, 10 bar CH_4, 1 bar CO, 293 K, 10 mL H_2O, 400 W Hg−Xe lamp	60	0.019	90.6	10
12[a]	6%Co-3%Pd /TiO_2	1500 mg, 50 mL/min CH_4, 50 mL/min CO_2, 423 K, 0.2 mL/min H_2O,	dual-reactor system	1	40	11
13	IrCuPd/ZSM-5	423 K, 40 mg, 20 mL H_2O, 30 bar CH_4, 10 bar CO, 5 bar O_2	5	13	0.044	12
14	Fe/ZSM-5	323 K, 30 mg, 10 mL 0.5 mol/L H_2O_2, 25 bar CH_4, 25 bar CO,	0.5	12.01	63.2	13

Supplementary Table 2. Comparative experiments of CH_4 conversion to acetic acid.

Entry	Reactants	Temperature (K)	Liquid products distribution
1	CH_4+H_2+O_2	463	CH_3OH, HCOOH
2	Without catalyst	463	n.d.
3	$FeCl_2$	463	n.d.
4	$Rh(NO_3)_3$	463	n.d.
5	$FeCl_2$ and $Rh(NO_3)_3$	463	n.d.
6	HCOOH+CO+O_2	463	n.d.
7	CH_3OH+CO+O_2	463	HCOOH
8	HCHO+CO+O_2	463	HCOOH

n.d. Not detected.

Supplementary Table 3. ICP of catalysts.

Catalyst	Metal Content (wt%)
Rh/ZSM-5	0.58
Fe/ZSM-5	0.26
RhFe/ZSM-5	Rh:0.55, Fe: 0.25
RhFe/ZSM-5 after reaction	Rh:0.53, Fe: 0.24
RhFe/ZSM-5-C	Rh:0.57, Fe: 0.28

Supplementary Table 4. Curvefit parameters for Rh K-edge and Fe K-edge EXAFS fitting parameters for Fe/ZSM-5, Rh/ZSM-5, and RhFe/ZSM-5.

Sample	Shell	S_0^2	σ^2/Å^2	d^e/Å	R/Å	ΔE	CN
Fe/ZSM-5 [a]	Fe-O_1	1.00	0.013±0.001	1.86	1.99±0.005	-4.45	3.9±0.23
	Fe-O_2	1.00	0.016±0.001	2.07	2.09±0.013	-4.45	0.1±0.03
Rh/ZSM-5 [b]	Rh-O_1	0.90	0.004±0.001	1.97	2.02±0.011	-3.70±1.72	4.5±0.49
	Rh-O_2	0.90	0.027±0.004	2.93	2.97±0.010	-3.70±1.72	0.9±0.11
	Rh-O_3	0.90	0.006±0.003	3.09	3.08±0.020	-3.70±1.72	1.0±0.31
RhFe/ZSM-5 [c,d]	Fe-O_1	1.00	0.018±0.002	1.86	2.03±0.004	8.26	4.0±0.67
	Fe-O_2	1.00	0.006±0.004	2.44	2.69±0.017	8.26	0.6±0.26
	Rh-O_1	1.00	0.004±0.001	1.97	2.02±0.007	-0.71±0.98	4.4±0.65
	Rh-O_2	1.00	0.003±0.002	2.93	3.04±0.018	-0.71±0.98	0.9±0.11
	Rh-O_3	1.00	0.005±0.005	3.09	3.21±0.038	-0.71±0.98	1.0±0.12

S_0^2: amplitude reduction factor;

σ^2: Debye-Waller factor;

d: set distance;

R: fitted distance;

CN: coordination number.

[a] Date ranges: $3.0 \leq k \leq 10$,$1.0 \leq R \leq 2.6$ Å. The number of variable parameters is 4, out of a total of 6.923 independent data points. R factor for these fits is 1.0%.

[b] Date ranges: $3.0 \leq k \leq 12.5$,$1.0 \leq R \leq 3.2$ Å. The number of variable parameters is 10, out of a total of 13.035 independent data points. R factor for these fits is 1.5%.

[c] Date ranges: $3.0 \leq k \leq 10$,$1.0 \leq R \leq 3.2$ Å. The number of variable parameters is 6, out of a total of 9.638 independent data points. R factor for these fits is 0.8%.

[d] Date ranges: $3.0 \leq k \leq 12.5$,$1.0 \leq R \leq 3.0$ Å. The number of variable parameters is 7, out of a total of 11.75 independent data points. R factor for these fits is 1.0%.

[e] The distances for Fe-O and Rh-O are from the crystal structure of FeO_2, Fe_2O_3, RhO_2 and RhO_3.

It was assumed that the Debye-Waller factor is the same for all the first-shell metal pairs (Rh-O, Fe-O) to minimize the number of fitting parameters.

Supplementary Table 5. The Mössbauer fitted parameters of the Fe-zeolite samples, obtained at 4.2 K.

Sample/ Treatment	IS ($mm \cdot s^{-1}$)	QS ($mm \cdot s^{-1}$)	Hyperfine field (T)	Γ ($mm \cdot s^{-1}$)	Phase	Spectral contribution (%)
A.	0.53	0.88	-	1.08	$Fe^{(III)}$-D[a]	18
^{57}Fe-ZSM-5	0.50	0.03	41.6*	0.94	$Fe^{(III)}$-PHS[b]	79
	1.38	3.37		0.72	Fe(II)[c]	3
B.	0.50	1.06	-	1.09	$Fe^{(III)}$-D	28
Rh^{57}Fe-ZSM-5	0.50	0.03	41.9*	0.82	$Fe^{(III)}$-PHS	70
	1.32	3.51		0.72	Fe(II)	2

Experimental uncertainties: Isomer shift: I.S. ± 0.02 mm s^{-1}; Quadrupole splitting: Q.S. ± 0.05 mm s^{-1}; Line width: Γ ± 0.05 mm s^{-1}; Hyperfine field: ± 0.2 T; Spectral contribution: ± 3%; *Average magnetic field; **a** Dimeric high-spin $Fe^{(III)}$-$Fe^{(III)}$ complexes; **b** Isolated (monomeric) $Fe^{(III)}$ ions (paramagnetic hyperfine splitting); **c** Isolated $Fe^{(II)}$ ions.

Supplementary Table 6. The Mössbauer fitted parameters of the Fe/ZSM-5 sample, obtained at 4.2 K.

Sample/ Treatment	IS ($mm \cdot s^{-1}$)	QS ($mm \cdot s^{-1}$)	Hyperfine field (T)	Γ ($mm \cdot s^{-1}$)	Phase	Spectral contribution (%)
A.	0.21	2.26	-	0.93	$Fe^{(IV)}$=O (I) cyan	26
0.2% ^{57}Fe-ZSM-5	0.14	2.28	48.1	0.68	$Fe^{(IV)}$=O (II) magenta	10
O_2/Ar	0.31	1.44	-	1.15	$Fe^{(III)}$-D[a] red	30
230 C, 20 bar, 2h	0.37	0.04	36.7*	0.70	$Fe^{(III)}$-PHS[b] blue	30
	1.36	3.18	-	0.72	Fe(II)[c] green	4
B.	0.18	2.13	-	0.88	$Fe^{(IV)}$=O (I)	19
0.2% ^{57}Fe-ZSM-5	0.14	2.06	48.9	0.74	$Fe^{(IV)}$=O (II)	11
O_2/CO/Ar	0.36	1.52	-	1.32	$Fe^{(III)}$-D	21
230 C, 20 bar, 2h	0.38	0.03	37.4*	0.71	$Fe^{(III)}$-PHS	34
	1.13	2.85	-	1.09	Fe(II)	15
C.	0.19	2.06	-	0.98	$Fe^{(IV)}$=O (I)	19
0.2% ^{57}Fe-ZSM-5	0.13	2.08	49.1	0.73	$Fe^{(IV)}$=O (II)	12
O_2/CO/CH_4	0.35	1.32	-	1.14	$Fe^{(III)}$-D	31
230 C, 20 bar, 2h	0.37	0.05	37.2*	0.72	$Fe^{(III)}$-PHS	31
	1.29	2.92	-	0.84	Fe(II)	7
D.	0.37	1.39	-	0.90	$Fe^{(III)}$-D	38
0.2% ^{57}Fe-ZSM-5	0.35	0.03	42.1*	0.79	$Fe^{(III)}$-PHS	46
O_2/CO/Ar/H_2O	1.29	2.70	-	0.98	Fe(II)	16
230 C, 20 bar, 2h						
E.	0.37	1.30	-	0.93	$Fe^{(III)}$-D	41
0.2% ^{57}Fe-ZSM-5	0.36	0.01	43.4*	0.74	$Fe^{(III)}$-PHS	47
O_2/CO/CH_4/H_2O	1.35	2.70	-	0.96	Fe(II)	12
230 C, 20 bar, 2h						

Experimental uncertainties: Isomer shift: I.S. ± 0.02 mm s^{-1}; Quadrupole splitting: Q.S. ± 0.05 mm s^{-1}; Line width: Γ ± 0.05 mm s^{-1}; Hyperfine field: ± 0.2 T; Spectral contribution: ±3%; *Average magnetic field; [a]Dimeric high-spin $Fe^{(III)}$-$Fe^{(III)}$ or $Fe^{(III)}$-$Fe^{(IV)}$ complexes; [b]Isolated (monomeric) $Fe^{(III)}$ ions (paramagnetic hyperfine splitting); [c]Isolated $Fe^{(II)}$ ions.

Supplementary Table 7. The Mössbauer fitted parameters of the Fe(Rh)/ZSM-5 sample, obtained at 4.2 K.

Sample/ Treatment	IS ($mm\cdot s^{-1}$)	QS ($mm\cdot s^{-1}$)	Hyperfine field (T)	Γ ($mm\cdot s^{-1}$)	Phase	Spectral contribution (%)
A. $Rh^{57}Fe$-ZSM-5	0.21	2.27	-	0.82	$Fe^{(IV)}$=O (I)	16
O_2/Ar	0.16	2.21	48.3	0.65	$Fe^{(IV)}$=O (II)	10
230 C, 20 bar, 2h	0.31	1.50	-	1.13	$Fe^{(III)}$-D[a]	43
	0.35	0.03	36.4*	0.75	$Fe^{(III)}$-PHS[b]	29
	1.36	3.23	-	0.74	Fe(II)[c]	2
B. 0.6% Rh	0.22	2.24	-	0.82	$Fe^{(IV)}$=O (I)	18
0.2% ^{57}Fe-ZSM-5	0.18	2.22	48.3	0.55	$Fe^{(IV)}$=O (II)	9
O_2/CO/Ar	0.32	1.61	-	1.26	$Fe^{(III)}$-D	31
230 C, 20 bar, 2h	0.32	0.04	37.2*	0.75	$Fe^{(III)}$-PHS	35
	1.26	3.09	-	0.99	Fe(II)	7
C. $Rh^{57}Fe$-ZSM-5	0.17	1.88	-	1.20	$Fe^{(IV)}$=O (I)	19
O_2/CO/CH_4	0.17	1.98	49.2	0.70	$Fe^{(IV)}$=O (II)	8
230 C, 20 bar, 2h	0.37	1.36	-	0.98	$Fe^{(III)}$-D	29
	0.38	0.04	38.6*	0.75	$Fe^{(III)}$-PHS	32
	1.32	2.81	-	0.99	Fe(II)	12
D. $Rh^{57}Fe$-ZSM-5	0.34	1.52	-	1.32	$Fe^{(III)}$-D	27
O_2/CO/Ar/H_2O	0.33	0.01	40.9*	0.97	$Fe^{(III)}$-PHS	70
230 C, 20 bar, 2h	1.32	3.18	-	0.74	Fe(II)	3
E. $Rh^{57}Fe$-ZSM-5	0.32	1.54	-	1.12	$Fe^{(III)}$-D	26
O_2/CO/CH_4/H_2O	0.36	0.01	41.5*	1.09	$Fe^{(III)}$-PHS	57
230 C, 20 bar, 2h	1.32	2.91	-	1.02	Fe(II)	17

Experimental uncertainties: Isomer shift: I.S. ± 0.02 mm s^{-1}; Quadrupole splitting: Q.S. ± 0.05 mm s^{-1}; Line width: Γ ± 0.05 mm s^{-1}; Hyperfine field: ± 0.2 T; Spectral contribution: ±3%; *Average magnetic field; [a]Dimeric high-spin $Fe^{(III)}$–$Fe^{(III)}$ or $Fe^{(III)}$–$Fe^{(IV)}$ complexes; [b]Isolated (monomeric) $Fe^{(III)}$ ions (paramagnetic hyperfine splitting); [c]Isolated $Fe^{(II)}$ ions.

Supplementary Table 8. The possible C-C coupling reaction pathway.

Reaction	Site
O_2 + $Fe^{(III)}$-OH → Fe $^{(IV)}$=O	Fe
O_2 + $Rh^{(II)}$-OH /$Rh^{(III)}$-OH → $Rh^{(III)}$=O	Rh
H_2O + Fe $^{(IV)}$=O → •OH + Fe $^{(IIII)}$-OH	Fe
$Rh^{(III)}$=O + CO →CO-$Rh^{(III)}$=O	Rh
CH_4 + CO-$Rh^{(III)}$=O→ •CH_3 + CO-$Rh^{(II)}$-OH	Rh
CO + •OH → •COOH	Rh / Fe
•CH_3 + •COOH → CH_3COOH	Rh / Fe
Side reaction:	
•CH_3 + •OH → CH_3OH	Rh / Fe
CO + *O → CO_2	Rh / Fe

Supplementary References

[1] Li, B. et al. Direct conversion of methane to oxygenates on porous organic polymers supported Rh mononuclear complex catalyst under mild conditions. Appl. Catal., B **293**, 120208, (2021).

[2] Qi, G. et al. Au-ZSM-5 catalyses the selective oxidation of CH_4 to CH_3OH and CH_3COOH using O_2. Nat. Catal. **5**, 45-54, (2022).

[3] Wu, B. et al. Fe binuclear sites convert methane to acetic acid with ultrahigh selectivity. Chem **8**, 1658-1672, (2022).

[4] Tang, Y. et al. Single rhodium atoms anchored in micropores for efficient transformation of methane under mild conditions. Nat. Commun. **9**, 1231, (2018).

[5] Shan, J., Li, M., Allard, L. F., Lee, S. & Flytzani-Stephanopoulos, M. Mild oxidation of methane to methanol or acetic acid on supported isolated rhodium catalysts. Nature **551**, 605-608, (2017).

[6] Narsimhan, K. et al. Methane to Acetic Acid over Cu-Exchanged Zeolites: Mechanistic Insights from a Site-Specific Carbonylation Reaction. J. Am. Chem. Soc. **137**, 1825-1832, (2015).

[7] Li, H. et al. Selective Methane Oxidation by Heterogenized Iridium Catalysts. J. Am. Chem. Soc. **145**, 769-773, (2023).

[8] Xie, Z. et al. Electrocatalytic Methane Oxidation to Ethanol via Rh/ZnO Nanosheets. J. Phys. Chem. C **125**, 13324-13330, (2021).

[9] Zerella, M., Kahros, A. & Bell, A. Methane oxidation to acetic acid catalyzed by Pd^{2+} cations in the presence of oxygen. J. Catal. **237**, 111-117, (2006).

[10] Dong, C. et al. Direct Photocatalytic Synthesis of Acetic Acid from Methane and CO at Ambient Temperature Using Water as Oxidant. J. Am. Chem. Soc. **145**, 1185-1193, (2023).

[11] Huang, W., Sun, W. Z. & Li, F. Efficient synthesis of ethanol and acetic acid from methane and carbon dioxide with a continuous, stepwise reactor. AIChE J. **56**, 1279-1284, (2009).

[12] Li, M. et al. Single-step selective oxidation of methane to methanol in the aqueous phase on iridium-based catalysts. Appl. Catal., B **292**, 120124, (2021).

[13] Wang, C.-W. et al. Oxidative carbonylation of methane to acetic acid on an Fe-modified ZSM-5 zeolite. Appl. Catal., B **329**, 122549, (2023).
